# Land expropriation, litigation, and reinterpretation of space in the genesis of the zoobotanical park of Museu Paraense Emílio Goeldi, 1895-1941

**DOI:** 10.1590/S0104-59702025000100066en

**Published:** 2026-02-09

**Authors:** Diego Leal, Nelson Sanjad

**Affiliations:** i Research fellow, Institutional Capacity-Building Program/Museu Paraense Emílio Goeldi. Belém – PA – Brazil. diego.leal1205@gmail.com; ii Professor, Graduate Program in Sociocultural Diversity/Museu Paraense Emílio Goeldi. Belém – PA – Brazil. nelsonsanjad@gmail.com

**Keywords:** Natural history museum, Botanical garden, Zoo, Historical heritage

## Abstract

This article analyzes the construction of the zoological and botanical gardens for Museu Paraense Emílio Goeldi, in Belém, northern Brazil. Opened in 1895 on a single property, the museum’s grounds were expanded over almost fifty years until they occupied the entire block – where it remains to this day. The research investigates the compulsory acquisition, with state and federal government support, of the neighboring properties, focusing on the political discourse formulated to justify the museum’s expansion and the way the affected parties resisted the acquisitions. As a result, the museum’s zoobotanical park is presented as a space in constant transformation, permeated by different meanings attributed by different social groups.

## On the relationship between republic, city, and museum

In the 1870s and 1880s, Brazil underwent some major political and economic changes. In its final years, the imperial regime was criticized for hindering the commercial development of the provinces due to political and tax centralization. It was believed that the high turnover of provincial presidents – appointees of Emperor Pedro II – was one factor behind the administrative discontinuities. These issues spawned criticism of the imperial government by provincial deputies, leaders of industry and commerce, and intellectuals in general (Costa, 1999; Santos, 2023).

The liberals – some strongly influenced by positivism – were keen to emphasize the importance of establishing a republic, arguing that the monarchy was a time of obscurity, when no significant progress was made in public education, science and technology investments, and the enhancement of the national economy (Alonso, 2002). In this sense, the advent of the republic in Brazil did not only represent a new political regime, but also meant the strengthening of regional elites dissatisfied with an overly centralized and oligarchic government, as well as an ideological construct whose symbols, allegories, and myths recreated the social imaginary, deconstructing any traces of the erstwhile monarchy (Carvalho, 1990; Ferreira, 2007; Farias, 2016).

Once the republic was proclaimed, educational, scientific, cultural, and public health institutions gained strategic importance as core priorities of the new government. The reasons for this were twofold: these institutions would help “reform,” “educate,” and “civilize” the people, as articulated in the manifesto of the new regime, and they would also serve as channels for disseminating the liberal-republican ideology to every level of Brazilian society. Accordingly, the institutionalization of science and the inclusion of scientists, naturalists, physicians, and intellectuals in government institutions gained a new boost as the new republic sought to gain legitimacy, prestige, and stability, while also meeting the interests of the new liberal elite. This same process also heralded the professionalization of public administration, strengthened the economy, and expanded the population’s access to education, cultural services, and healthcare (Almeida, 2003; Sá, 2006; Sanjad, 2010; Arraes, 2022).

All this took place at an extremely propitious time thanks to the federal pact, which gave the former provinces administrative autonomy, and the tax reform, which meant a significant proportion of tax revenues was decentralized. In the Amazonian state of Pará, for example, this period coincided with the “rubber economy,” which had begun in the late 1800s, based on the extraction of latex from certain tree species and the export of unprocessed rubber to European and North American markets. The state’s revenues in the early republican years rose from 24,544,569$ in 1889 to 32,306,545$ in 1890, rising even more sharply in 1891, when they reached 50,019,310$; i.e., a 100% increase vis-a-vis 1889 (Silva, 1996).

These data indicate just how much some states and municipalities could benefit from the new political and fiscal regimes, setting the stage for bold plans to reinvent whole cities and their cultural institutions, as was seen in the Amazonian cities of Belém and Manaus (Dias, 1999; Sarges, 2010). In Belém, for example, the first decades of the republican period saw the construction of boulevards*,* squares, and monuments for the leisure and enjoyment of the population, in addition to the installation of basic urban services such as water distribution, sewage collection, and public transportation, as well as expanded access to electricity (Coelho, 2002; Sarges, 2010).

Behind the reforms and improvements introduced to Belém’s public spaces was a desire to observe the principles of public health and hygiene, which called for the construction of parks and provision of street cleaning and basic sanitation services. However, these public services were limited to the most central neighborhoods, where the wealthier segments of the population lived. According to an analysis by Sarges (2010, p.152), the idea of the republic was what catalyzed all these transformations, as argued in the following passage:

The dynamizing action of embellishing the city was associated with the economy and demographics, but also with the aesthetic values of an upwardly mobile class (rubber barons, merchants, and farmers) and the need to provide certain segments of the city’s population with security and housing, in addition to putting into practice the positivist idea of progress emphasized by the new republican regime.

The urbanization of Belém in the first decades of the republic revealed a controversial side in its socio-spatial segregation and the effects this had on the lives of affected populations – a phenomenon also called social exclusion, which results from capitalist relations by producing unequal access to the production, circulation, and exchange of social wealth. The demolition of poor housing in the city center, as well as the introduction of a building code that effectively excluded the lower classes, radically altered the appearance of the city and enabled the emergence of new economic groups that owned land and capital (Coimbra, 2024).

It is based on this approach that we analyze one of the most important urban projects of the first republican governments in Belém: the renovation of Museu Paraense (the Pará Museum), created in 1866 and renamed Museu Paraense de História Natural e Etnografia (the Pará Museum of Natural History and Ethnography) in 1894. The first governor of Pará, Lauro Sodré, who served between 1891 and 1897, was responsible for initiating this institutional project, hiring Emil Goeldi (1859-1917) as the museum’s director in 1894. The plans the two men drew up had two major influences: the urban redevelopments in Belém, especially the Europeanization of the city’s landscape and the introduction of parks and public leisure areas, as well as what has been termed the natural history museum movement, which had gained ground internationally since the mid-1800s. Thanks to this network, museums could create new organizational and administrative structures or improve existing ones. It also encouraged them to engage in the networked production of scientific knowledge, the formation of collections, and public education.

In her analysis of the creation and trajectory of natural history museums in nineteenth-century Brazil, Lopes (1997) emphasizes the internal dynamics inherent to each one, as well as their organizational structures, leadership teams and employees, their ties with other museums at home and abroad, their contributions to education, and the impetus they gave to the study of the natural sciences. In this regard, Gualtieri (2008) argues that the growing specialization and scientific rigor imposed on natural history museums in the late nineteenth century were evidence of the influence of the theories of evolution, which determined the choices made by each director in terms of research activities. We should not lose sight of the fact that notwithstanding their differences, these research hubs had a common essence, which was encapsulated in the endeavors by part of the Brazilian intelligentsia to ensure that the scientific activities undertaken in the country were attuned with the prevailing international standards.

**Figure 1:**
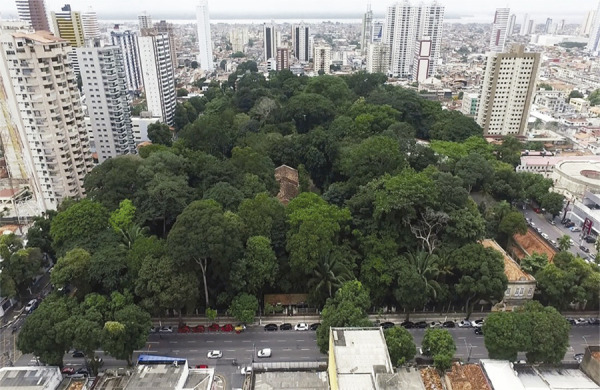
: Zoobotanical park of Museu Paraense Emílio Goeldi, in Belém, Pará, photograph by Dronetech, 2016 (courtesy of the Social Communication Service, Museu Paraense Emílio Goeldi)

Sanjad’s (2010) study of Museu Paraense reveals how close politics and science were under Emperor Pedro II (1840-1889) and in the early republican years (1889-1930). His study covers several aspects of the formation of this museum, including the development of a scientific agenda and the introduction of professional specialization, as well as the creation of new headquarters for the museum at the dawn of the republican years. To this end, the administrations of Lauro Sodré (1891-1897), José Paes de Carvalho (1897-1901), and Augusto Montenegro (1901-1909) introduced some authoritarian measures, including the compulsory eviction of residents from land in areas considered to be of public utility by the state government. This process was halted in 1908 and only completed during the administration of José da Gama Malcher (1935-1943), when the headquarters of Museu Paraense took over an entire block, between what are now Avenida Magalhaes Barata (formerly Estrada da Independência), Travessa Nove de Janeiro, Avenida Gentil Bittencourt (formerly Estrada da Constituição), and Avenida Alcindo Cacela (formerly Travessa Vinte e Dois de Junho) (Figure 1).

To better explore this subject, which has not yet been developed in the historiography, despite its centrality to the history of this listed heritage, we have divided this article into three topics. In the first, we analyze the justifications for the acquisition of a property that was relatively far from the city center to house the new headquarters of Museu Paraense. In the second section, we analyze the first phase of the museum’s expansion, between 1895 and 1908. In the third, we look at the second phase, between 1933 and 1943, during the authoritarian government of Getúlio Vargas (1930-1945). In the concluding section, we present a table listing the property owners who were forced to vacate and sell their land for the museum and the location of their lots, as well as some possible avenues of research stemming from this study.

The sources used in the research consist of administrative documents from the Guilherme de La Penha Archive of Museu Paraense Emílio Goeldi, the museum’s own institutional reports, and plans prepared by museum employees and state government functionaries kept in the Pará public archives (Arquivo Público do Estado do Pará). Additionally, state government reports, communiqués, and official gazettes were particularly important in helping us understand the administrative and legal procedures and the way funding was negotiated in the state legislature for the acquisition of land and buildings and for the construction of nurseries, animal enclosures, monuments, and lakes in the grounds.

Finally, it is worth highlighting the importance of the probate records kept at the Amazon Memory Center (Centro de Memória da Amazônia) of the Federal University of Pará (Universidade Federal do Pará), which helped us identify the socioeconomic profile of some of the residents whose land was the target of compulsory acquisition processes. These documents provided details about the assets, prestige, and family alliances of the former owners: a wealth of information that enabled us to reconstruct, to some extent, the way in which these individuals engaged with and displayed agency in the eviction and acquisition processes. Newspapers, laws, executive orders, printed images, and photographs maintained in the aforementioned institutions were also consulted.

### Development of the zoobotanical park begins

In 1891, as part of the new republican government’s efforts to overhaul public education, it established a new headquarter for the Museu Paraense, on Rua São João (nowadays Rua João Diogo), in the center of Belém, next to the old Liceu Paraense (Sanjad, 2010). Under the direction of Ernesto Acton, a taxidermy specialist, the museum received many donations of live animals, which were kept in precarious conditions. In 1893, Acton contacted the state governor, Lauro Sodré, with the suggestion that botanical and zoological gardens be created as part of the museum. This idea was subsequently executed under the administration of Emil Goeldi, who succeeded Acton in March 1894. On June 2 of that year, a decree issued by Sodré defined the new regulations for the museum, which included zoological and botanical gardens as “annexes” to the museum (Regulamento..., 1894).

To fulfill its new remit, Goeldi felt the museum needed “a more appropriate building with sufficient capacity to allow for the development and expansion of the collections,” since the old building’s size and state of repair inconsistent with what the governor had in mind for the “new museum” (Goeldi, 1895, p.218). After carefully examining several properties in the city, the director and his staff expressed great interest in *rocinha* n.22 (literally “little farm #22”), owned by Bento José da Silva Santos, on Estrada da Independência (now Avenida Governador Magalhães Barata).

Like its country homes (*chácaras*), Belém had had *rocinhas*, or smallholdings, since the late eighteenth century: large, landscaped properties in the suburbs that typically had vegetable gardens and orchards, as well as livestock for the household’s own consumption. At the height of the rubber boom, grander houses started to be built, combining the comforts of city living with the rural landscape (Soares, 1996; Soares, 2008). As Sodré and Goeldi showed interest in acquiring Silva Santos’s *rocinha*, one of the largest in the city, he decided to ask for Rs.120:000$000 (denominated in *contos de réis ouro*), including a downpayment of Rs.20:000$000 upon the signing of the purchase contract. This sum was paid, and the remaining debt was negotiated with the state treasury. Goeldi (1895, p.218) made the following observation about the property:

The layout of the interior of the building, solid structure, dimensions, careful conservation, and pleasing aspect, were the many other factors in its favor, given that the smallholding is located among plots not small that allow for the construction of those annexes to the museum, which the government has in mind, namely a modest Zoological Garden and a Botanical Garden, it was decided that among the public and private properties presently available in the city, none offered the same combination of advantages and desirable qualities.

It is worth noting that, notwithstanding the architectural qualities of the building, the location – “among plots not small” – was also associated with an old leisure area in Belém, as there had been a public garden there in the 1870s. This garden had stretched from Travessa Nove de Janeiro to the current-day Travessa Quatorze de Março, behind the Basilica of Our Lady of Nazareth, with a frontage of more than 500 meters. It was a private entertainment area that visitors had to pay to access. The sources consulted suggest that it was first opened as the “Olympic Circus” (Circo Olympico) in late 1870, then renamed “Mythological Garden” (Jardim Mitológico) on June 4, 1871 (O Liberal..., 20 jul. 1871; Diário de Belém, 15 out. 1882; Diário de Notícias, 4 dez. 1894, 7 dez. 1894). However, it was short-lived: by the late 1870s, the immense plot of land had been carved up and sold off, lot by lot. Bento José da Silva Santos had purchased six lots, choosing the largest, on Estrada da Independência, for his villa, which he constructed in 1879. The rest of former Mythological Garden was purchased by different people in various ways.

The acquisition and occupation of Silva Santos’s property and the construction of the “annexes” of Museu Paraense as of 1895 bore no direct relation to the Mythologial Garden itself, but only with the availability of land from the estate of the garden’s former owner. In the 1890s, Silva Santos was the sole owner of one quarter of the block now occupied by the museum’s grounds. Therefore, the new museum premises in no way represented an attempt to recapture the experience of the former park; it was simply one of the many projects pursued as part of the municipal development policy as of 1890, especially the creation of new public parks and leisure areas.

Belém city hall was directly involved in the planning of the museum’s new premises through Sociedade Zeladora do Museu Paraense (the museum’s supporters’ society), created in 1896 and chaired by the mayor and Baron of Marajó, José Coelho da Gama e Abreu. The aim of the new society was to protect, preserve, and promote the museum, making it a center for research and public education. Goeldi counted on the support of the local political elite to realize his plans, making the supporters’ society an intermediary between the museum, the city hall, the government, and the state legislature (Goeldi, 1897a; Marajó, 1897).

**Figure 2:**
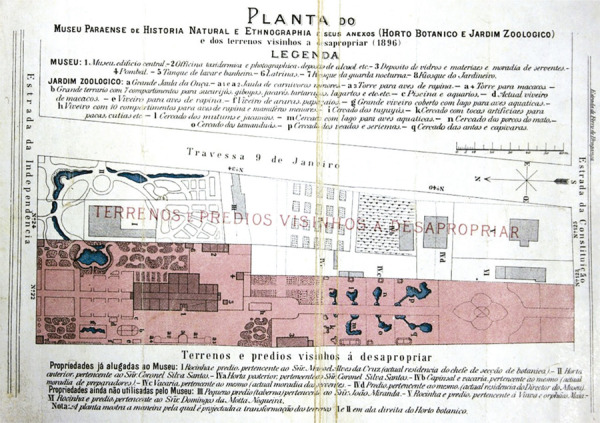
: Plan of Museu Paraense de História Natural e Etnografia in 1896, showing the property acquired for the installation of the museum (in pink), and the interventions made and planned for it and the other plots on Travessa Nove de Janeiro (Goeldi, 1897b)

From 1895 onwards, the buildings and land purchased from Silva Santos were totally refurbished and adapted to house the museum’s collection and furniture (Suescun Florez, Sanjad, Okada, 2018). The plot of land covering 14,962m^2^ extended sideways, dividing the block into two parts, from Estrada da Independência to Estrada da Constituição (now Avenida Gentil Bittencourt), with the difference that the front was wider than the back. During the government of Paes de Carvalho, the lots owned by Silva Santos and the other four on Travessa Nove de Janeiro, all of which had been developed, were declared of public utility by the state of Pará based on law 499 of May 15, 1897. This was equivalent to half of the block, as can be seen in the plan published by Goeldi in 1897 (Figure 2).

From 1896 onwards, the state legislature approved funding that enabled the planned interventions to be made to the property, namely, exhibition spaces inside the building, two “annexes,” laboratories, workshops, and employees’ housing. The botanical garden and the zoo were both designed for public education purposes, as the aim was to create an “attractive school for learning about the works of Amazonian nature,” but on a modest scale, with no pretention of “wishing to imitate the great gardens and botanical gardens of overseas” (Goeldi, 1895, p.220).

The transformation of the grounds, which included the construction of animal enclosures, nurseries, tree-lined promenades, borders, plantations, lakes, buildings, and monuments, has been analyzed elsewhere, including the identification of the first animals and plants kept there. The public’s response to the museum’s new premises has also been thoroughly documented (Sanjad, 2006, 2008, 2009, 2010; Machado, 2010; Sanjad et al., 2012; Suescun Florez, 2015; Suescun Florez, Sanjad, Okada, 2018). What is worth noting here is that Goeldi’s role went beyond designing and coordinating the development of the buildings and grounds; he also negotiated agreements with nearby residents to get them to rent out their homes and lands and accept the compensation offered by the government to vacate their properties altogether. According to Goeldi (1897b, p.258-259), the agreements signed with these homeowners were designed to prevent “arbitrary fluctuations:” the possibility of their filing demands or requests for further compensation, which could interfere with the plans for the grounds. It would appear that these agreements therefore served the interests of the government and the museum, but, as we will see, they sometimes ran counter to the wishes of the landowners themselves.

### First phase of expansion of the zoobotanical park

After the enactment of law 499 in 1897, which paved the way for the acquisition of the lots on Travessa Nove de Janeiro (see Figure 2), the state government set up a land valuation commission composed of two engineers, Henrique Santa Rosa (director of public works) and Victor Maria da Silva (an inspector from the municipal water company, Águas de Belém), tasked with examining the state of the properties and valuing them. Goeldi was adamant that the commission should begin by inspecting the properties of Manoel Alves da Cruz and João Ribeiro de Miranda (plots I and III, respectively, on the drawing in Figure 2), since the botanical garden was planned to occupy their land, making its vacancy a priority (Goeldi, 1897b, p.3).

Goeldi’s arguments to justify the acquisition of Manoel Alves da Cruz’s property were authoritarian: that he had failed to honor agreements and commitments, that he was making “demands” and “charged exorbitant rent” for a property in such poor condition, with “buckled and cracked” walls and “rotten floors,” as well as being insalubrious and unhygienic for anyone who did not have the “constitution of a batrachian [amphibian]” (Goeldi, 1897b, p.259; Goeldi, 1900, p.3-4). In his view, the only thing of any real value was “the land and not the dwelling,” which it would make more sense to demolish than to renovate (Goeldi, 1900, p.4).

Cruz turned down the government engineers offer of payment for him to vacate his property, saying he felt it was below its market value. However, he did rent his house and grounds to the museum, although this also gave rise to legal disputes with the government. In August 1898, the Pará state government gazette, *Diário Oficial do Estado*, published a letter from Cruz asking the chief treasury official to authorize a rent hike for his property from Rs.200$000 to Rs.300$000 per month, which, he claimed, would bring it into line with the amount the museum paid to the other property owners whose land it rented (Diário Oficial…, 14 ago. 1898, p.326). A few days later, Cruz received a reply stating that the rent increase he had requested had not been approved by either the museum director or the state governor, since eviction proceedings were already underway and it was not “in the interests of the State” to increase the amount paid for the property (Diário Oficial…, 27 set. 1898, p.605).

Cruz’s rejection of the government’s offer prompted displeasure on the part of the authorities. In March 1900, he appealed to the state secretary to intervene in the conflict with Goeldi. The lease had expired and had not been renewed by Cruz, who had asked Goeldi to vacate the property and return the keys. When Goeldi refused to hand over the keys, Cruz sent another letter, this time to Governor Paes de Carvalho, asking for his house to be returned to him so he could live there with his family (Secretaria do Estado do Pará, 27 jul. 1900, p.1).

Goeldi’s strategy was to buy time until the compulsory purchase proceedings were concluded in his favor. This only occurred on February 1, 1901, during the administration of Augusto Montenegro: Museu Paraense was granted the immediate and definitive possession of Manuel Alves da Cruz’s smallholding and João Miranda de Ribeiro’s ranch (Montenegro, 1901). However, ownership of Cruz’s property was only officially transferred on August 28, 1901, after Virgílio Cardoso de Oliveira, Secretary of State for Justice, Interior, and Public Education, notified the director of the museum that the house could now be used (Secretaria de Estado da Justiça..., 28 ago. 1901).

The exact reason for the delay is unknown, but the appeals filed by Cruz likely prolonged the conflict somewhat, not to mention the use he made of the local press to publicize his criticism of the government’s action. Cruz did not receive his compensation in cash, but in a promissory note from the state of Pará, in the amount of Rs.28:500$000. Not only was this amount lower than he felt he was owed, but Cruz also had to face repeated delays in the payment of his rent between July 1898 and August 1901. Although he publicly voiced his disgruntlement with the irregularity of these monthly payments, he was ignored by the state authorities and the museum director (Diário Oficial..., 8 jul. 1898, p.73, 25 ago. 1898, p.391, 10 jan. 1900, p.50, 16 mar. 1900, p.521, 6 abr. 1900, p.40, 9 maio 1900, p.229, 31 ago. 1901, p.299).

Goeldi (1904, p.4), in turn, said he was “relieved” that this acquisition had finally happened, since the delay in its completion had been a “source of frustration and annoyance.” Although the building itself served no purpose for the museum, the land was essential for the planned botanical gardens. Once the plot was acquired, the house was demolished and an eye-catching water tower, known as the “small castle,” was built on the site, along with several plant nurseries and bird cages. What had been the kitchen garden, at the back of the house, was planted with latex-producing trees; that is, it was taken over by the Botany Department to musealize living specimens (Huber, 1901, p.104).

As for the ranch owned by João Ribeiro de Miranda, on number 34, Travessa Nove de Janeiro, the owner accepted the valuation presented to him, which simplified the process: all that remained was for the agreement signed by both parties to be notarized. However, Goeldi (1901, p.106-107) did not want to wait for the formalities, fearing that things “might get complicated,” so he recommended to the government that the property be vacated with “all due haste.” There was a particular reason why Goeldi wanted to acquire that relatively small 560m^2^ plot of land, occupied by a single tavern, which quite possibly sold cheap alcoholic beverages: he felt it was “aesthetically repugnant” (Goeldi, 1904, p.4) and a “source of iniquity,” jeopardizing the discipline he wished to impose on the museum staff, especially its gardeners and zookeepers. The house was described as being “in ruins” and was valued at Rs.8:000$000 by the government experts.

According to Goeldi (1906, p.469), the ranch itself was “a cattle farm located on the old grasslands,” which gives us a sense of how that land was used. It could have been an old property used to raise dairy cattle for milk production, which was a very common activity on the outskirts of Belém at the time. Ribeiro de Miranda, who died shortly after the land was acquired, left the compensation he was owed to his widow, Antônia Maria de Jesus (Inventário de João..., 27 set. 1898). The tavern was soon demolished and replaced with a Swiss chalet-style residence for Emílio Goeldi’s cousin, Andreas Goeldi, who was employed as the botanical garden superintendent, as well as a depot for the glassware and other materials used to preserve the collections and housing for the museum’s housekeeping and maintenance staff (Goeldi, 1906, p.469). This change was hugely beneficial for the institution, insofar as the supervisor’s house was strategically located in the east section of the grounds, enabling them to be monitored, while also functioning as a gatehouse to control the access of workers and tradespeople (Goeldi, 1904, p.9).

For purposes of comparison, it is worth analyzing the process by which the lots belonging to Bento José da Silva Santos were acquired. According to the sources consulted, Silva Santos was an important businessman in the second half of the nineteenth century. His business interests, which included agriculture, extractivism, trade, and industry, were based in the town of Chaves, on Marajó Island, in municipalities along the Pará coast, on Arapiranga Island (facing Belém, but part of the municipality of Barcarena), and in Belém itself. According to the probate records, Silva Santos’s family business, Silva Santos & Filhos (Silva Santos & Sons), had the following assets: 12 plots of land and properties in Belém, farms, developments, cattle, warehouses, boats, a steam launch, machinery, and equipment, as well as investments in rubber tree plantations. Upon his death on May 23, 1908, aside from his company’s assets, Silva Santos left behind real estate assets in his own name valued at 539:000$000 (denominated in *contos de réis*) (Inventário de Bento..., 23 maio 1908).

Silva Santos, like many other rubber barons, livestock famers, and industrialists, held various public offices: a requirement for the reproduction of his fortune and for forging alliances with other landowners. It was important for wealth to be combined with other factors, particularly a name and surname, which was achieved through marital arrangements (Cunha, 2008). Silva Santos married his daughter Maria da Silva Santos to a congressman, Pedro Leite Chermont, who belonged to the family that dominated the local political scene in the nineteenth century and throughout the early republican years (1889-1930). As Cancela (2011, p.353) explains, alliances of this type often “worked as a strategy for preserving wealth, as well as for restricting the entrance of individuals with no lineage into the kinship network.” Such strategies – restricted to the world of the Pará elite – would also perpetuate the surnames of the families in question.

When Silva Santos’s land on Estrada da Independência and Travessa Nove de Janeiro became the target of compulsory acquisition orders, he was a member of the municipal council, where he served for many years. His property was vacated for Museu Paraense in 1895, and it was also in this year that construction began for a mansion and houses for his married children on four lots he owned on Estrada de Nazaré, near Praça da República. Palacete Silva Santos, or the Silva Santos mansion, later renamed Palacete Faciola, now houses Museu da Imagem e do Som (the Museum of Image and Sound). It is a classic example of the types of buildings constructed by the elite who controlled economic production and public services in Belém in the late 1800s (Derenji, 2023; Marques, Baleixe, 2023).

It would appear that Silva Santos, unlike Manoel Alves da Cruz and João Ribeiro de Miranda, was in the enviable position of being able to set the rent for his properties and determine their purchase value. This is borne out in a telegram he sent to the state secretariat in November 1898, stating that he wanted to raise the rent of one of the plots used by Museu Paraense from Rs.300$000 to Rs.400$000 a month (Diário Oficial…, 4 nov. 1898, p.197). On July 19, 1899, Augusto Olímpio de Souza, the state secretary, informed Goeldi that, pursuant to a “communication” received from Silva Santos, he had decided to raise the monthly rent for his properties to Rs.400$000, retroactive to January 1 of that year, “until the acquisition already authorized takes place” (Secretaria do Estado do Pará, 19 jul. 1899).

Interestingly, Manoel Alves da Cruz had made exactly the same request three months earlier, but he had been refused. The justification given by the government was based on precisely the same argument it had used to grant approval of Silva Santos’s request. In Cruz’s case, the rent hike would not be granted because the property was in the process of being acquired; in Silva Santos’s case, the hike was justified by the fact that the property would soon be acquired. It is clear, then, how differently Silva Santos was treated, even though his property was in a similar situation to that of Cruz, namely, soon to be acquired for the museum.

The disparity between the treatment of Cruz and Silva Santos does not end here. While Cruz received a promissory note as compensation for his property, on August 30, 1899, upon authorization given by the Public Treasury, Silva Santos was paid Rs.26:000$000: six more than stipulated in the initial agreement (Diário Oficial…, 8 jan. 1899, p.45). Silva Santos then made his other lots adjacent to lot number 22 available for purchase by the state government for Rs.32:000$000. The government immediately accepted the offer without any negotiation. On September 22, 1899, less than a month after the first payment was made, Museu Paraense took definitive possession of all the land that had belonged to Silva Santos (Goeldi, 1901, p.106-107).

After the lots on Travessa Nove de Janeiro were incorporated into Museu Paraense, the first steps were taken to acquire the properties on Estrada da Constituição. In official correspondence (Ofício 483) dated November 27, 1899, the Public Treasury was notified that house no. 123, owned by Diogo Pinto da Silva, had been rented for Rs.250$000 a month (lot VI in Figure 2, whose owner in 1897 had been Domingos da Motta Nogueira) (Secretaria do Estado do Pará, 27 nov. 1899). However, just a few weeks later, on January 5, 1900, decree 794 was sanctioned by the government authorizing the Treasury to make credit of Rs.8:880$208 available as compensation for the acquisition of that same property (Diário Oficial…, 5 jan. 1900, p.30). On January 10, 1900, Goeldi personally notified the resident, who agreed to vacate the property to make way for some of the museum’s researchers.

Eight years later, the state governor, Augusto Montenegro, acquired another house on Estrada da Constituição, this time for Rs.20:000. It belonged to widow Maia and her orphaned children (lot V in Figure 2). Curiously, however, our research shows that years before, this very same lot had been offered to the government for Rs.70:000, but had been turned down because the price was considered exorbitant. Negotiations had dragged on until 1908, when it was sold for far less than the owner had originally asked. This was the last lot to be incorporated into the museum along the perimeter between Silva Santos’s *rocinha* and Travessa Nove de Janeiro (Montenegro, 1908, p.175).

Even though some major developments had been carried out in the museum since 1897, Goeldi repeatedly complained that there was not enough space. In 1899, he made an urgent call for a “second building equal to or larger than the existing one,” since the rooms and corridors of the main building were already crammed full with the collections. Erecting another building would resolve some of the problems he was facing. This justification was then forwarded to the State Congress with a request to acquire the rest of the block: the properties on Travessa Vinte e Dois de Junho (now Avenida Alcindo Cacela), especially a house belonging to a doctor, Miguel José de Almeida Pernambuco, which Goeldi had earmarked for the botany and geology departments (Goeldi, 1901, p.107-108).

The decision to acquire Doctor Miguel Pernambuco’s property marked the beginning of the museum’s expansion along Travessa Vinte e Dois de Junho. Although Goeldi made an initial request early in 1900 by means of an official letter to the State Treasury inspector (Diário Oficial..., 14 jan. 1900, p.80), it was not until 1903 that Governor Montenegro (1902, p.40) secured the funding needed to commence this new phase.

In 1903, negotiations began for the acquisition of a smallholding (*rocinha*) owned by Hermínio Cardozo da Cunha Coimbra on number 42, Estrada da Independência, adjacent to Silva Santos’s. Cunha Coimbra had inherited this property from his mother, Libéria Maria Joaquina Corrêa Coimbra, who had died in 1900 (Inventário de Libéria..., 17 out. 1900). She had been married to a Portuguese merchant, Antônio Cardoso da Cunha Coimbra, with whom she had had three children: José, Hermínio, and Joaquim. The oldest of the three, José, was appointed executor of the will, with the consent of his father, who was still alive. Hermínio, who lived in Taubaté, São Paulo, negotiated his share with the museum on a visit to Belém in 1903, although he only received his payment of Rs.12:600$000 in 1905. The two other sons, José and Joaquim, also inherited land on the same block, but this was only taken over by the museum decades later, as we will see in the next section.

**Figure 3:**
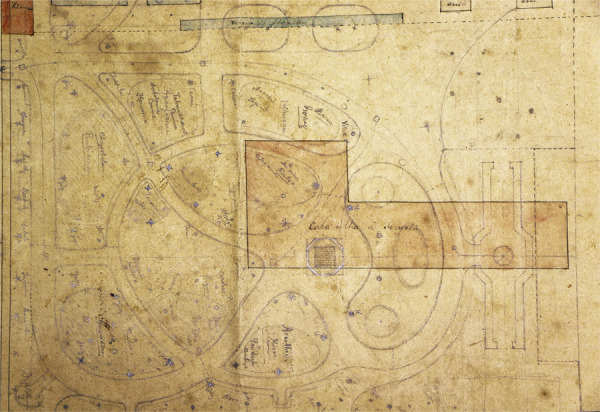
: Layout of the garden to be created on the land formerly owned by Hermínio Cardozo da Cunha Coimbra (property lines marked in pink), designed by Jacques Huber, 1907 (Pará Public Archives, Belém)

A 1907 plan kept in the Pará public archives that was drawn up by Jacques Huber, Goeldi’s successor as director of the museum, shows the detailed drawing for the garden to be created on Hermínio’s land, including borders, promenades, and monuments (Figure 3). The drawing also identifies the plant species to be introduced and the way the new space would be integrated into the grounds. This new garden was opened in 1908, together with a bust of the museum’s founder, the naturalist Domingos Soares Ferreira Penna (1818-1888), from the southeastern state of Minas Gerais, sculpted by Rodolpho Bernardelli (Sanjad, 2010).

This continuous expansion of the museum’s grounds demonstrates the level of support it received from the state government over three administrations, spanning 15 years. Every year, it received supplementary credit of Rs.30:000$000 for its expansion and improvement, a sum that rose to Rs.50:000$000 in 1901, covering half of its annual budget (Goeldi, 1904). Throughout this period, Museu Paraense was the public institution that received the biggest investment from the state government (Sanjad, 2010).

### The second phase of expansion of the zoobotanical park

**Figure 4:**
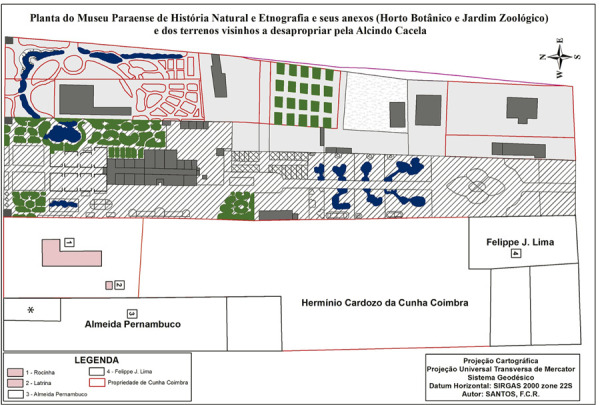
: Zoned survey plan of the zoobotanical park of Museu Paraense de História Natural e Etnografia (the Pará Museum of Natural History and Ethnography), produced in 1901, based on the original plan prepared by the surveyor Aristides Pereira Leão, preserved in the Pará Public Archives, in Belém. This plan provides a visual representation of the second stage of expansion of the museum’s grounds (prepared by Fernando Carlos in 2020 using ArcGis 10.7)

In 1901, Aristides Pereira Leão, a surveyor from the Pará Department of Public Works, conducted a land and topographic survey of the block where Museu Paraense was being installed, on a scale of 1:400 (Figure 4). The resulting survey plan, commissioned by Goeldi, is preserved in the Pará State Public Archives and, like the drawing in Figure 3, remained unknown until this research was conducted. The plan shows the interventions made in the block since the grounds were first developed in 1895, as well as the property lines of the lots on Travessa Vinte e Dois de Junho, which were not included in the plan published by Goeldi in 1897 (see Figure 2). Due to the poor state of conservation of the 1901 drawing, a digitalized version was produced to enable its analysis.

The lots already incorporated into the museum by 1901 appear in light gray in Figure 4. In the upper right-hand corner, the property of widow Maia, which she vacated in 1897, was only acquired in 1908. The lots on Travessa Vinte e Dois de Junho, in white, were still privately owned, including those owned by the Cunha Coimbra family. Hermínio Cardozo da Cunha Coimbra’s property, negotiated in 1903 and acquired in 1905, is marked in pink. It should be noted that this was only a small fraction of the land the family owned on that block.

The lots on Travessa Vinte e Dois de Junho were only acquired during the administration of Carlos Estevão de Oliveira (1930-1945), during the years of the Getúlio Vargas dictatorship. Although the plan Oliveira executed was drawn up by Goeldi at the end of the nineteenth century, the political context was different. The first properties to be acquired for the installation of the zoobotanical park were all funded by the state government, which also covered the costs of creating borders, animal enclosures, monuments, lakes, and buildings. According to Sanjad (2010), these developments were integrated into the broader range of educational and urban reforms pursued by the first republican leaders of Pará. Museu Paraense was regarded as both an institution for the education of the citizenry, evidence of the gentility of the local elite, and a vehicle for the promotion of Belém, which already had an international presence thanks to the rubber trade.

During the Vargas years, Museu Paraense was resignified in line with the nationalist, modernist discourse of the day. In his analysis of this, Figueiredo (2001) draws attention to the expansion of the federal government’s policies and Vargas’s alliances with local political groups – a process that once again made Museu Paraense a focus of political attention. Goeldi’s plans for the museum, put on the back burner by the Pará state administrators in the 1920s in the face of steeply declining revenues from rubber, were reinstated and rescaled to align them with Vargas’s priorities and those of his local acolytes. A full analysis of the complexity of this subject falls beyond the scope of this article, but can be found in the works of Leal (2023, 2024), who investigated Oliveira’s efforts to get Museu Paraense integrated into national public policies, and Andrade (2024), who focuses on Vargas’s speeches on the Amazon and state interventions in the region, especially in the fields of health and nutrition.

Despite the changed circumstances, one thing is clear: the time was ripe for Goeldi’s plans to be revived; namely, to expand the grounds to Travessa Vinte e Dois de Junho and to secure support for the expansion of the institution’s infrastructure, this time not from the bankrupt state coffers, but from the federal government, which demonstrated receptiveness to regional demands and willingness to invest in science and technology. In 1930, there were still eight lots to be incorporated into the museum: four belonging to José and Joaquim da Cunha Coimbra, two belonging to Suzana Evangelina Autran, one to Felippe Lima, and one to Agostinho de Menezes Monteiro (see Figure 4).

Carlos Estevão de Oliveira restarted the process of acquiring the lots adjacent to the museum in 1933, after an agreement had been signed for the federal government to transfer funds to the state of Pará for the expansion of the botanical garden, the creation of an orchid house and a palm grove, and the construction of an ichthyology laboratory and tanks for fish breeding. The latter two items were demands made by the federal government itself, as it wanted to integrate Museu Paraense into the National Fish Farming Program, coordinated at the time by the Northeast Fish Farming Technical Commission. This program prioritized investments in research into the reproduction of Amazonian fish to stock reservoirs in the Northeast, combining science and food production policies with drought mitigation measures (Oliveira, 2022; Leal, 2024).

As a result of this strategic project, the state government set about acquiring the two houses belonging to Suzana Autran. At the beginning of the century, these had been a single property, but she had divided it into two after the death of her husband, Agostinho Autran, moving into one house and renting out the other (Inventário de Agostinho..., 27 jan. 1933). In the same year that she received her inheritance, Suzana was pressured to negotiate with the government of Pará, as her land was classified as a public utility for its value as part of the museum grounds. She was ultimately paid Rs.16:000$000 for the property (Inventário de Agostinho..., 27 jan. 1933), which was used for the palm grove and orchid house, opened in 1934 (Dyer, 1945, p.3).

Also in 1934, Museu Paraense acquired some other lots using funds raised from the trade in Amazonian ornamental fish (Leal, 2024). The lots in question were the ones owned by the Cunha Coimbra family and Felippe Lima, on the corner of Travessa Vinte e Dois de Junho and Avenida Gentil Bittencourt (formerly Estrada da Constituição) (Suescun Florez, 2015). Construction of the museum’s fish farm began on this land as part of the plans to expand the “capacity of the zoology department” (Barata, 1944, p.172).

These acquisitions gave the museum greater control over the block, although there was still a large plot of land that belonged to the Cunha Coimbra family and the house owned by Agostinho de Menezes Monteiro on Avenida da Independência (formerly Estrada da Independência), marked with an asterisk on the 1901 plan (Figure 4) because it had not yet been built at that time. Thanks to federal government funds, these remaining lots could finally be acquired. Between 1935 and 1941, the federal government gave the museum five separate grants, totaling Cr$510,000.00 (denominated in *cruzeiros*), of which Cr$320,000.00 went to covering the costs of the property purchases (Museu Paraense..., 11 nov. 1942, 14 nov. 1942).

Agostinho Monteiro was the protagonist of the last (tense) compulsory acquisition. According to Castro (13 jun. 2013), he was a doctor, professor, and politician. He was the administrator of the Hospital of the Third Order of the Franciscans (Hospital da Ordem Terceira de São Francisco) and also ran the Pará Faculty of Medicine and Surgery, as well as teaching at the school of trade (Escola Prática do Comércio) and the Pará school of agronomy and veterinary science (Escola de Agronomia e Veterinária do Pará). He served as a state deputy from 1926 to 1930 and a federal deputy in 1934 for Frente Única Paraense (Pará United Front), a political party that opposed the federally instated governor, Magalhães Barata, and his political supporters. In the early 1940s, Monteiro acquired the large plot of land belonging to José and Joaquim da Cunha Coimbra, where he planned to build a rangy two-story house with 48 rooms. This residential complex, designed and developed by architect J.E. Levi, would have a garden with palm-lined central and lateral avenues. He negotiated the mortgage for the building with the government-owned bank Caixa Econômica Federal, as well as a social security establishment, Instituto de Aposentadoria, and a bank and mortgage lender, Lar Brasileiro. In early 1941, the only thing that remained to be done was for the mayor of Belém, Abelardo Conduru, to sign the papers granting authorization for work to begin. If built, the Marajó Building would have taken up almost a third of the block that now houses the museum and its grounds (Folha do Norte, 16 abr. 1941, p.2, 26 abr. 1941, p.2).

Oliveira managed to prevent the construction of the building, which Monteiro had announced in the local press, by sending a technical opinion to the state governor, José Malcher. On March 31, 1941, his department embargoed the work on the grounds that the building would prevent the expansion of the museum. A decree was passed requiring ownership of all the properties on Avenida da Independência and Travessa Vinte e Dois de Junho that did not yet belong to Museu Paraense to be transferred to the state, which would pay compensation to their present owners based on technical valuation. The mayor, accepting the state-level decision, notified Monteiro that he could not sign off on the project, as the property would have to be sold to the state to expand the museum (Folha do Norte, 16 abr. 1941, p.2).

On May 12, 1941, Malcher authorized the compulsory acquisition of Agostinho Monteiro’s house and the large plot of land on Travessa Vinte e Dois de Junho. Monteiro’s planned estate never got off the drawing board, and Monteiro entered into negotiations over his compensation with the state government, even though it was paid with federal funds. By 1943, there were already laboratories, lakes, and tanks for the intensive breeding of various species of Amazonian turtles and fish on his land – all integrated into the activities of the Northeast Fish Farming Technical Commission – as well as temporary tanks for ornamental fish, which the museum bred and sold at the time (Leal, 2024). The house on Avenida da Independência and Miguel de Almeida Pernambuco’s former residence were both maintained. These, together with Silva Santos’s former home and another house on 40, Travessa Nove de Janeiro (lot IVd on the 1897 plan, see Figure 2), used as the museum director’s residence until the 1950s, are the only buildings that remain from the properties prior to their acquisition for the museum.

## Final considerations

During the administrations of Emil Goeldi (1894-1907), Jacques Huber (1907-1914), and Carlos Estêvão de Oliveira (1930-1945), twenty lots belonging to 11 individuals or families were acquired for the construction of the zoobotanical park of Museu Paraense, in a process that began in 1895 and ended in 1941, when the last properties were officially listed as the museum’s assets. Under Goeldi, 11 plots were acquired and occupied by the museum between 1895 and 1905; under Huber, there was just one acquisition, in 1908, of a property occupied in 1897; and under Oliveira there were eight compulsory acquisitions between 1933 and 1941. Bento José da Silva Santos and Agostinho Monteiro were the property owners who had the most land acquired for the museum: six and three lots, respectively.

Table 1 shows the list of landowners, the location of their properties, and the date of their acquisition, which should not be confused with the date on which they were vacated to make way for the museum, as sometimes lengthy negotiations ensued to determine the value of the compensation to be paid by the state government. The data contained in this table were obtained from all the different stages of the compulsory acquisition processes, documented in a range of sources such as Museu Paraense reports, the Pará state gazette (*Diário Oficial do Estado*), probate records*,* newspapers, and landscape and architectural drawings.

**Table 1:** : List of owners of land and properties taken by the state for the construction of the zoological and botanical gardens of Museu Paraense Emílio Goeldi between 1895 and 1941

Owner	Location	No. of lots	Date of acquisition
Bento José da Silva Santos	Estrada da Independência, no. 22 Estrada da Independência (no number) Travessa Nove de Janeiro (no number) Travessa Nove de Janeiro (no number) Travessa Nove de Janeiro (no number) Travessa Nove de Janeiro, no. 40	6	1895-1899
Diogo Pinto da Silva (identified as Domingos da Mota Nogueira in 1897)	Estrada da Constituição, no. 123	1	1899
Manoel Alves da Cruz	Estrada da Independência, no. 24	1	1901
João Ribeiro de Miranda	Travessa Nove de Janeiro, no. 34	1	1901
Miguel José de Almeida Pernambuco	Estrada da Independência, ?	1	1903-1905
Herminio Cardozo da Cunha Coimbra	Estrada da Independência, no. 42	1	1905
Widow Maia and orphans	Estrada da Constituição, no. 125	1	1908
Suzana Evangelina Autran	Travessa Vinte e Dois de Junho, no. 439 Travessa Vinte e Dois de Junho, no. 445	2	1933
José and Joaquim Cardozo da Cunha Coimbra	Travessa Vinte e Dois de Junho, ?	2	1934
Felippe J. Lima	Estrada da Constituição, ?	1	c.1941
Agostinho Menezes Monteiro, Julia Menezes Monteiro, and the heirs of Maria Dias Brito	Estrada da Independência, no. 172 Travessa Vinte e Dois de Junho, no.182-184 Travessa Vinte e Dois de Junho, no. 186	3	1941

Source: elaborated by the authors.

During the museum’s initial phase of expansion, those landowners who put up a fight or refused compensation for their properties were publicly stigmatized by Goeldi, who dubbed them “obstacles to progress” and found fault with their homes. Goeldi was prejudiced against the less prestigious properties and the local way of life. Whether Huber shared such views cannot be gauged as he did not express any such opinions in his reports. Although he was also in favor of further compulsory acquisitions to expand the botanical gardens, he was less authoritarian towards the museum’s neighbors. The suspension of further acquisitions during his tenure was mainly due to budgetary constraints as of 1910 on the back of the decline in rubber prices, triggering administrative issues, late wage payments, budget cuts, staff layoffs, and fewer expeditions, publications, and acquisitions of scientific collections (Sanjad, 2019).

The suspension of the land acquisition program can also be explained by the increase in the value of the land around the museum – a direct consequence of the urban infrastructure improvements made to enable the museum itself, such as the paving of the nearby roads, the introduction of piped water, the expansion of the electricity supply, and nighttime security guards, all of which Goeldi commented on in his annual reports. As the neighborhood gained value, the plans to take ownership of the remaining properties became unfeasible for the state budget. The last acquisitions were only possible thanks to federal funds received during the Getúlio Vargas administration and brokered by Carlos Estevão de Oliveira.

Goeldi, Huber, and Oliveira justified the expansion of Museu Paraense on the grounds that it needed more space for its collections and more research infrastructure, with new offices and laboratories. They also disparaged the museum’s neighbors and advocated for state intervention for the creation of a space of education and social utility in the city. The high visitor numbers to the grounds, as discussed by Sanjad (2006, 2010), were key to the state governors’ and museum directors’ rhetoric in favor of further interventions in the block, securing funding from the State Congress, and garnering political support for the negotiations with the residents, which often became public. Later, the political and financial support the museum received during Getúlio Vargas’s first term as president catalyzed a new wave of compulsory purchases after a twenty-five-year hiatus with no investments by the state government. The involvement of the museum (and its director, Carlos Estevão de Oliveira) in federal government projects and actions in the Amazon region, such as the Northeast Fish Farming Technical Commission, the National Historical and Artistic Heritage Service, and the Supervisory Council for Artistic and Scientific Expeditions in Brazil, assured the museum relative stability in the state government bureaucracy in the 1930s and 1940s as compared with the 1920s (Leal, 2023, 2024).

Evidently, Museu Paraense was not the only Brazilian institution to be transformed or created after Brazil became a republic. Analyses have been made of similar processes at several long-standing scientific institutions, such as the Ouro Preto School of Mines (Escola de Minas de Ouro Preto) (Carvalho, 1978), the National Museum (Museu Nacional) (Lopes, 1997), the Paulista Museum (Museu Paulista) (Alves, 2001), the Rio de Janeiro Botanical Garden (Jardim Botânico do Rio de Janeiro) (Bediaga et al., 2008; Heizer, 2012), and the Oswaldo Cruz Institute (Instituto Oswaldo Cruz) (Benchimol, 1990), as well as medical and scientific societies and schools of medicine. Heizer and Videira (2010) edited a volume to discuss the professional trajectories and transformations wrought in educational and research establishments by the republican regime. However, rarely do such studies focus on the relationship between these institutions and their host cities. We might ask, for example, what impact the transfer of the National Museum to Boa Vista Palace in 1892 had on the Rio de Janeiro neighborhood São Cristóvão, or how the installation of the Paulista Museum in the Ipiranga Monument in 1893 affected the lives of the surrounding population. Similar questions could be asked of later periods, such as the consequences of the urban interventions that gave rise to the National Historical Museum in 1922 and the creation of the Imperial Museum in Petrópolis in 1940.

Studies in the field of health and medicine seem to devote more attention to the relationship between the institutionalization of science and its impacts on urban life, as evidenced in the research of Benchimol (1999), Almeida (2003), and Mota (2005). This was the approach prioritized in our research: to understand, on a micro scale, how transformations in the political and scientific fields affected the lives of people living in the vicinity of Museu Paraense from 1895 onwards. These people were directly impacted by the decisions of Lauro Sodré and Emil Goeldi. Both were committed to creating a model institution that had a strong symbolic significance for the local political elites and was at the same time strongly connected to international scientific networks, as demonstrated elsewhere (Lopes, 1997; Sanjad, 2010). This article shows that, in addition to a more institutional approach, focused on the organizational structure, budgets, scientific work, employees, national and international exchanges, government relations, etc., another perspective is possible that looks at the social collective physically connected to the institution and indelibly affected by it.

## Data Availability

Not deposited in a data repository.

## References

[B1] ALMEIDA Marta de (2003). República dos invisíveis: Emílio Ribas, microbiologia e saúde pública em São Paulo (1898-1917).

[B2] ALONSO Angela (2002). Ideias em movimento: a geração 1870 na crise do Brasil-Império.

[B3] ALVES Ana Maria de Alencar (2001). O Ipiranga apropriado: ciência, política e poder. O Museu Paulista, 1893-1922.

[B4] ANDRADE Rômulo de Paula (2024). Amazônia na era do desenvolvimento: saúde, políticas e destruição (1930-1966).

[B5] ARRAES Jonas (2022). Tão longe e tão distante: a presença de Carlos Gomes na belle époque de Belém do Pará.

[B6] BARATA Joaquim de Magalhães Cardoso (1944). Relatório ao senhor presidente da República pelo interventor federal coronel Joaquim de Magalhães Cardoso Barata.

[B7] BEDIAGA Begonha (2008). Jardim Botânico do Rio de Janeiro, 1808-2008.

[B8] BENCHIMOL Jaime (1999). Dos micróbios aos mosquitos: febre amarela e a revolução pasteuriana no Brasil.

[B9] BENCHIMOL Jaime (1990). Manguinhos, do sonho à vida: a ciência na belle époque.

[B10] CANCELA Cristina Donza (2011). Casamento e família em uma capital amazônica (1870-1920).

[B11] CARVALHO José Murilo (1990). A formação das almas: o imaginário da República no Brasil.

[B12] CARVALHO José Murilo (1978). A Escola de Minas de Ouro Preto: o peso da glória.

[B13] CASTRO Ribamar A história no Diário Oficial: Agostinho Menezes Monteiro.

[B14] COELHO Geraldo Mártires (2002). No coração do povo: o monumento à República em Belém, 1891-1897.

[B15] COIMBRA Adriana (2024). Do combate aos cortiços à habitação social: um estudo sobre história urbana e moradia popular em Belém do Pará (1890-1940).

[B16] COSTA Emília Viotti (1999). Da Monarquia à República: momentos decisivos.

[B17] CUNHA Marly Solange Carvalho (2008). "Matutos" ou astutos? Oligarquia e coronelismo no Pará Republicano (1897-1909).

[B18] DERENJI Jussara (2023). Palacete Faciola.

[B19] (1882). DIÁRIO DE BELÉM.

[B20] (1894). DIÁRIO DE NOTÍCIAS.

[B21] (1894). DIÁRIO DE NOTÍCIAS.

[B22] (1901). DIÁRIO OFICIAL do Estado do Pará.

[B23] (1900). DIÁRIO OFICIAL do Estado do Pará.

[B24] (1900). DIÁRIO OFICIAL do Estado do Pará.

[B25] (1900). DIÁRIO OFICIAL do Estado do Pará.

[B26] (1900). DIÁRIO OFICIAL do Estado do Pará.

[B27] (1900). DIÁRIO OFICIAL do Estado do Pará.

[B28] (1900). DIÁRIO OFICIAL do Estado do Pará.

[B29] (1899). DIÁRIO OFICIAL do Estado do Pará.

[B30] (1898). DIÁRIO OFICIAL do Estado do Pará.

[B31] (1898). DIÁRIO OFICIAL do Estado do Pará.

[B32] (1898). DIÁRIO OFICIAL do Estado do Pará.

[B33] (1898). DIÁRIO OFICIAL do Estado do Pará.

[B34] (1898). DIÁRIO OFICIAL do Estado do Pará.

[B35] DIAS Edinea Mascarenhas (1999). A ilusão do fausto: Manaus, 1890-1920.

[B36] DYER Emília (1945). Relatório botânico. Arquivo Guilherme de La Penha, Fundo Museu Paraense Emílio Goeldi, Gestão Carlos Estêvão de Oliveira.

[B37] FARIAS William Gaia (2016). A construção da República no Pará (1886-1897).

[B38] FERREIRA Sylvio Mário Puga (2007). Federalismo, economia exportadora e representação política: o Amazonas na República Velha (1889-1914).

[B39] FIGUEIREDO Aldrin Moura, Faulhaber Priscila, Toledo Peter Mann (2001). Conhecimento e fronteira: história da ciência na Amazônia.

[B40] (1941). FOLHA DO NORTE.

[B41] (1941). FOLHA DO NORTE.

[B42] GOELDI Emílio Augusto (1906). Relatório apresentado ao Sr. Dr. secretário da Justiça, Interior e Instrução Pública, referente ao ano de 1902, pelo diretor do Museu. Boletim do Museu Goeldi de História Natural e Etnografia.

[B43] GOELDI Emílio Augusto (1904). Relatório sobre o Museu, relativo ao ano de 1901, apresentado ao Exmo. Sr. Dr. secretário de Estado da Justiça, Interior e Instrução Pública pelo Dr. Emílio Augusto Goeldi, diretor do mesmo museu. Boletim do Museu Goeldi de História Natural e Etnografia (Museu Paraense).

[B44] GOELDI Emílio Augusto (1901). Relatório apresentado ao Exmo. Sr. Dr. José Paes de Carvalho, governador do Estado do Pará, pelo diretor do Museu Paraense de História Natural e Etnographia. Ano de 1899. Boletim do Museu Paraense de História Natural e Etnografia.

[B45] GOELDI Emílio Augusto (1900). Relatório apresentado ao Exmo. Sr. Dr. Paes de Carvalho, governador do Estado do Pará, pelo diretor do Museu Paraense de História Natural e Etnografia. Boletim do Museu Paraense de História Natural e Etnografia.

[B46] GOELDI Emílio Augusto (1897a). Discurso proferido pelo diretor do Museu por ocasião da instalação da Sociedade Zeladora do Museu Paraense em 6 de novembro de 1896. Boletim do Museu Paraense de História Natural e Etnografia.

[B47] GOELDI Emílio Augusto (1897b). Relatório apresentado ao Exmo. Sr. Dr. Lauro Sodré, governador do Estado do Pará, pelo diretor do Museu Paraense. Boletim do Museu Paraense de História Natural e Etnografia.

[B48] GOELDI Emílio Augusto (1895). Relatório apresentado pelo diretor do Museu Paraense ao Sr. Dr. Lauro Sodré, governador do Estado do Pará. Boletim do Museu Paraense de História Natural e Etnografia.

[B49] GUALTIERI Regina Cândida Ellero (2008). Evolucionismo no Brasil: ciência e educação nos museus (1870-1915).

[B50] HEIZER Alda (2012). João Barbosa Rodrigues: um naturalista entre o Império e a República. Revista Brasileira de História da Ciência.

[B51] HEIZER Alda, VIDEIRA Antônio Augusto Passos (2010). Ciência, civilização e república nos trópicos.

[B52] HUBER Jacques (1901). Apontamentos sobre o movimento do Museu Paraense no ano de 1898. Boletim do Museu Paraense de História Natural e Etnografia.

[B53] INVENTÁRIO DE AGOSTINHO Autran (1933). Centro de Memória da Amazônia, 2ª Vara Civil, Cartório Odon Rhossard.

[B54] José da Silva Santos INVENTÁRIO DE BENTO (1908). Centro de Memória da Amazônia, 4ª Vara Cível, Cartório Leão.

[B55] INVENTÁRIO DE JOÃO Ribeiro de Miranda (1898). Centro de Memória da Amazônia, 14ª Vara Civil, Cartório Sarmento, Cx. 14-20-B.

[B56] INVENTÁRIO DE LIBÉRIA Maria Joaquina Corrêa Coimbra (1900). Centro de Memória da Amazônia, 1ª Vara Civil, Cartório Santiago.

[B57] LEAL Diego Rodrigo Guimarães (2024). Ciência, nação e região na Era Vargas: o caso do Museu Paraense Emílio Goeldi (1930-1945).

[B58] LEAL Diego Rodrigo Guimarães (2023). Trajetórias profissionais e instituições científicas na Era Vargas: a atuação política e científica de Carlos Estêvão de Oliveira (1930-1941). Revista Brasileira de História da Ciência.

[B59] LOPES Maria Margaret (1997). O Brasil descobre a pesquisa científica: os museus e as ciências naturais no século XIX.

[B60] MACHADO Diego Ramon Silva (2010). A "lição de coisas": o Museu Paraense e o ensino de História Natural (1889-1900).

[B61] MARAJÓ Barão de (1897). Discurso proferido pelo Exmo. Sr. barão de Marajó. Boletim do Museu Paraense de História Natural e Etnografia.

[B62] MARQUES Fernando, BALEIXE Haroldo, Derenji Jussara (2023). Palacete Faciola.

[B63] MONTENEGRO Augusto (1908). Mensagem dirigida em 7 de setembro de 1908 ao Congresso Legislativo do Pará pelo Dr. Augusto Montenegro, governador do Estado.

[B64] MONTENEGRO Augusto (1902). Mensagem dirigida em 7 de setembro de 1902 ao Congresso Legislativo do Pará pelo Dr. Augusto Montenegro, governador do Estado.

[B65] MONTENEGRO Augusto (1901). Mensagem do governador Augusto Montenegro ao Congresso do Estado do Pará em 10 de setembro de 1901.

[B66] MOTA André (2005). Tropeços da medicina bandeirante: medicina paulista entre 1892 e 1920.

[B67] MUSEU PARAENSE Emílio Goeldi (1942). Ofício do diretor ao interventor José da Gama Malcher. Arquivo Guilherme de La Penha, Fundo Museu Paraense Emílio Goeldi, Gestão Carlos Estêvão de Oliveira, Série Correspondência.

[B68] MUSEU PARAENSE Emílio Goeldi (1942). Ofício do diretor ao delegado fiscal do Pará, Alexandre Castro Filho. Arquivo Guilherme de La Penha, Fundo Museu Paraense Emílio Goeldi, Gestão Carlos Estêvão de Oliveira, Série Correspondência.

[B69] (1871). O LIBERAL do Pará.

[B70] OLIVEIRA Emanuel Rodolpho Moura Batista (2022). História da Comissão Técnica de Piscicultura do Nordeste: Helmintologia, Limnologia, Ictiologia e Botânica (1932-1945).

[B71] REGULAMENTO do Museu Paraense (1894). Boletim do Museu Paraense de História Natural e Etnografia.

[B72] SÁ Dominichi Miranda de (2006). A ciência como profissão: médicos, bacharéis e cientistas no Brasil (1895-1935).

[B73] SANJAD Nelson (2019). Nimuendaju, a Senhorita Doutora e os 'etnógrafos berlinenses': rede de conhecimento e espaços de circulação na configuração da etnologia alemã na Amazônia no início do século XX. Asclepio.

[B74] SANJAD Nelson (2010). A coruja de Minerva: o Museu Paraense entre o Império e a República, 1866-1907.

[B75] SANJAD Nelson (2009). Emílio Goeldi (1859-1917): a ventura de um naturalista entre a Europa e o Brasil.

[B76] SANJAD Nelson (2008). A revitalização do Parque Zoobotânico do Museu Goeldi: em busca de uma nova relação com o público. Museologia e Patrimônio.

[B77] SANJAD Nelson (2006). A "simpatia do povo" pelo Museu Paraense: raízes históricas. Musas.

[B78] SANJAD Nelson (2012). Documentos para a história do mais antigo jardim zoológico do Brasil: o Parque Zoobotânico do Museu Goeldi. Boletim do Museu Paraense Emílio Goeldi. Ciências Humanas.

[B79] SANTOS Roberg (2023). Do Grão-Pará à Amazônia: a invenção da região amazônica frente à centralização do Império brasileiro.

[B80] SARGES Maria de Nazaré (2010). Belém: riquezas produzindo a belle époque (1870-1912).

[B81] SECRETARIA DE ESTADO DA JUSTIÇA, Interior e Instrução Pública (1901). Ofício ao diretor do Museu Paraense. Arquivo Guilherme de La Penha, Fundo Museu Paraense Emílio Goeldi, Gestão Emílio Goeldi, Série Correspondência, Subsérie Correspondência Passiva.

[B82] SECRETARIA DO ESTADO DO PARÁ (1900). Ofício ao diretor do Museu Paraense. Arquivo Guilherme de La Penha, Fundo Museu Paraense Emílio Goeldi, Gestão Emílio Goeldi, Série Correspondência, Subsérie Correspondência Passiva.

[B83] SECRETARIA DO ESTADO DO PARÁ (1899). Ofício ao diretor do Museu Paraense. Arquivo Guilherme de La Penha, Fundo Museu Paraense Emílio Goeldi, Gestão Emílio Goeldi, Série Correspondência, Subsérie Correspondência Passiva.

[B84] SECRETARIA DO ESTADO DO PARÁ (1899). Ofício ao diretor do Museu Paraense. Arquivo Guilherme de La Penha, Fundo Museu Paraense Emílio Goeldi, Gestão Emílio Goeldi, Série Correspondência, Subsérie Correspondência Passiva.

[B85] SILVA Moacir Fecury Ferreira da (1996). Do regional ao nacional: Pará (1895-1914).

[B86] SOARES Karol Gillet (2008). As formas de morar na Belém da belle époque (1870-1910).

[B87] SOARES Roberto de La Rocque (1996). Vivendas rurais do Pará: rocinhas e outras (do século XIX ao XX).

[B88] SUESCUN FLOREZ Lilian (2015). O modo expositivo dos museus de natureza: análise comparativa entre a exposição da coleção viva de flora do Parque Zoobotânico do Museu Paraense Emílio Goeldi e a representação da Região Amazônica do Jardim Botânico do Rio de Janeiro.

[B89] SUESCUN FLOREZ Lilian, SANJAD Nelson, OKADA Wanda (2018). Construção do espaço museal: ciência, educação e sociabilidade na gênese do Parque Zoobotânico do Museu Goeldi (1895-1914). Anais do Museu Paulista.

