# Supplementary material for: Land expropriation, litigation, and reinterpretation of space in the genesis of the zoobotanical park of Museu Paraense Emílio Goeldi, 1895-1941
Source: Hist Cienc Saude Manguinhos. 2026 Feb 9;32:e2025066. doi: 10.1590/S0104-59702025000100066en (PMC12885484; doi:10.1590/S0104-59702025000100066en)
Supplement: Translation

# Translation: Expropriação, litígio e ressignificação espacial na gênese do parque zoobotânico do Museu Paraense Emílio Goeldi, 1895-1941

**DOI:** 10.1590/S0104-59702025000100066

**Authors:** Diego Leal, Nelson Sanjad

**Affiliations:** i Pesquisador, Programa de Capacitação Institucional/Museu Paraense Emílio Goeldi. Belém – PA – Brasil. diego.leal1205@gmail.com; ii Professor, Programa de Pós-graduação em Diversidade Sociocultural/Museu Paraense Emílio Goeldi. Belém – PA – Brasil. nelsonsanjad@gmail.com

**Keywords:** Museu de história natural, Jardim botânico, Zoológico, Patrimônio histórico

## Abstract

O artigo analisa a construção do Parque Zoobotânico do Museu Paraense Emílio Goeldi, em Belém-PA. Inaugurado em 1895, a partir de uma única propriedade, o parque foi expandido ao longo de quase cinquenta anos, até ocupar todo o quarteirão onde hoje se encontra. A pesquisa enfatizou a expropriação, promovida pelos governos estadual e federal, dos antigos moradores daquela área – sobretudo o discurso político formulado para justificar a ampliação do parque. Acompanharam-se as formas de resistência tecidas nesse contexto, mediadas por diversos sujeitos. Como resultado, o Parque Zoobotânico é apresentado como um espaço em constante transformação, permeado de diferentes significados atribuídos por diferentes grupos sociais.

## Para pensar a relação entre República, cidade e museu

Nas décadas de 1870 e 1880, o Brasil passou por importantes mudanças políticas e econômicas. A fase final do regime imperial foi criticada por impedir o desenvolvimento comercial das províncias devido à centralização política e tributária. Acreditava-se que a troca frequente dos presidentes de províncias, nomeados por dom Pedro II, contribuía para a descontinuidade administrativa. Essas questões geraram críticas ao governo imperial por deputados provinciais, por representantes dos setores industrial e comercial e por intelectuais em geral (Costa, 1999; Santos, 2023).

Os liberais, parte dos quais era profundamente influenciada pelo positivismo, faziam questão de enfatizar a importância do processo de implantação da República, afirmando que o regime monárquico representava um momento de obscuridade, sem avanços significativos referentes à instrução pública, aos investimentos na área científica e tecnológica e à valorização da economia nacional (Alonso, 2002). Nesse sentido, o advento da República no Brasil não representou tão somente um novo regime político, mas significou o fortalecimento das elites regionais insatisfeitas com um governo demasiadamente centralizado e oligárquico, e também um constructo ideológico cujos símbolos, alegorias e mitos recriavam o imaginário social, desconstruindo os traços deixados pela monarquia (Carvalho, 1990; Ferreira, 2007; Farias, 2016).

Proclamada a República, as instituições educacionais, científicas, culturais e de saúde pública ganharam importância estratégica, isto é, foram alçadas a uma posição central no novo governo por dois fatores: seriam capazes de “reformar”, “instruir” e “civilizar” o povo, conforme o projeto político estabelecido pelo novo regime, assim como seriam os veículos capazes de disseminar a ideologia liberal-republicana em todas as camadas da sociedade brasileira. Destarte, o processo de institucionalização da ciência e de inserção de cientistas, naturalistas, médicos e intelectuais em instituições governamentais foi significativamente acelerado e ampliado, com a finalidade de conferir legitimidade à República, garantindo-lhe prestígio, estabilidade e assegurando os interesses da nova elite liberal, e também de profissionalizar a administração pública, fortalecer a economia e ampliar o acesso da população à instrução e aos serviços culturais e de saúde (Almeida, 2003; Sá, 2006; Sanjad, 2010; Arraes, 2022).

Isso ocorreu em um momento extremamente favorável, em função do pacto federativo, que deu autonomia administrativa às antigas províncias, e da reforma tributária, que manteve nas províncias parte significativa da arrecadação de impostos. No Pará, por exemplo, desde finais do século XIX, esse momento coincidiu com a “economia da borracha”, baseada na extração do látex de espécies arbóreas gomíferas e na exportação de borracha bruta para os mercados europeu e norte-americano. A receita do estado nos primeiros anos da República evoluiu de 24.544.569$, em 1889, para 32.306.545$, em 1890, dando um salto ainda mais acentuado em 1891, quando atingiu o patamar de 50.019.310$, ou seja, cresceu 100% em relação a 1889 (Silva, 1996).

Esses dados nos indicam o quanto estados e municípios beneficiaram-se enormemente do novo regime político e do novo modelo fiscal, criando as condições para que fosse realizado um ousado projeto de reinvenção de cidades e instituições culturais, como foram os casos de Belém e Manaus (Dias, 1999; Sarges, 2010). Em Belém, por exemplo, foram construídos, nas primeiras décadas republicanas, *boulevards*, praças e monumentos, voltados para o lazer e o deleite da população, além de instalados serviços urbanos básicos, como distribuição de água, coleta de esgoto, ampliação da rede de energia elétrica e do transporte público (Coelho, 2002; Sarges, 2010).

O programa de reformas e melhorias do espaço urbano de Belém buscava atender, acima de tudo, aos princípios higienistas e às práticas salubristas, caracterizados pela construção de áreas verdes e pelos serviços públicos de limpeza urbana e saneamento básico. Tais serviços públicos, contudo, ficaram circunscritos aos bairros mais centrais, onde a população com maior poder aquisitivo residia. De acordo com a análise de Sarges (2010, p.152), a ideia de República foi o elemento catalisador que propiciou todas essas transformações, como se observa no seguinte trecho:

A ação dinamizadora de embelezamento do perfil desta cidade estava associada à economia, à demografia, mas também aos valores estéticos de uma classe em ascensão (seringalistas, comerciantes e fazendeiros) e à necessidade de se dar a determinados segmentos da população da cidade segurança e acomodação além da colocação em prática da ideia positivista de progresso enfatizada pelo novo regime republicano.

A urbanização da cidade de Belém, nas primeiras décadas republicanas, mostrou seu lado polêmico na segregação socioespacial e nos seus efeitos na vida das populações atingidas, fenômeno também chamado de exclusão social e que resulta das relações capitalistas ao produzir acesso desigual aos meios de produção, circulação e troca da riqueza social. A remoção de cortiços e habitações populares do Centro da cidade, assim como a exigência de um padrão construtivo não acessível a camadas mais baixas, provocou uma radical transformação do perfil estético da cidade e viabilizou a emergência de novos grupos econômicos detentores do solo e do capital (Coimbra, 2024).

É com base nessa abordagem que analisamos uma das mais importantes realizações urbanas forjadas pelos primeiros governos republicanos em Belém: a reforma do Museu Paraense, criado em 1866, rebatizado como Museu Paraense de História Natural e Etnografia, em 1894. Esse projeto institucional foi implantado a partir da gestão do primeiro governador do Pará, Lauro Sodré (1891-1897), responsável pela contratação de Emílio Goeldi (1859-1917) para a direção do museu, em 1894. O projeto que ambos desenvolveram alinhava-se, por um lado, às intervenções urbanas feitas em Belém, sobretudo a europeização da paisagem e a implantação de áreas verdes e de lazer na cidade; por outro lado, vinculou-se ao chamado movimento dos museus de história natural, que vinha se consolidando internacionalmente desde meados do século XIX. Essa rede de museus tornou possível a criação e/ou a requalificação do perfil organizacional e administrativo das instituições museológicas, que passaram a direcionar seus esforços para a produção em rede de conhecimentos científicos, formação de coleções e instrução pública.

Lopes (1997), ao analisar a criação e a trajetória dos museus de história natural no Brasil do século XIX, enfatizou as dinâmicas internas próprias de cada um deles, sua organização, seus diretores e funcionários, as relações estabelecidas com outros museus no país e no exterior, as contribuições ao ensino e o impulso que deram ao estudo das ciências naturais. A esse respeito, Gualtieri (2008) argumentou que a crescente especialização e o rigor científico impostos aos museus de história natural em finais do século XIX eram indícios das influências herdadas das teorias evolucionistas e evidenciadas pelas opções de cada diretor no direcionamento dado às atividades de pesquisa. Não podemos perder de vista que, apesar da diversidade institucional, tais núcleos de pesquisa convergiam para um ponto em comum, sintetizado no esforço de parte da intelectualidade brasileira para vincular as atividades científicas desenvolvidas no país aos padrões internacionais em voga.

O estudo de Sanjad (2010) sobre o Museu Paraense revela a estreita relação entre política e ciência no Segundo Reinado e na Primeira República. O autor analisou vários aspectos relacionados ao processo de formação desse museu, incluindo o desenvolvimento de uma agenda científica e da especialização profissional. Também foi estudada a gênese da nova sede erigida para o museu no alvorecer da Primeira República. Com essa finalidade, algumas medidas autoritárias foram aplicadas durante as gestões de Lauro Sodré (1891-1897), José Paes de Carvalho (1897-1901) e Augusto Montenegro (1901-1909), entre as quais se incluem a expropriação e a remoção dos moradores da área considerada de utilidade pública pelo governo do estado. Esse processo foi interrompido em 1908 e concluído apenas no governo de José da Gama Malcher (1935-1943), quando a sede do Museu Paraense pôde ocupar todo o quarteirão localizado entre as atuais avenida Magalhaes Barata (antiga estrada da Independência), travessa Nove de Janeiro, avenida Gentil Bittencourt (antiga estrada da Constituição) e avenida Alcindo Cacela (antiga travessa Vinte e Dois de Junho) (Figura 1).

**Figura 1:** : Parque Zoobotânico do Museu Paraense Emílio Goeldi, Belém, Pará, fotografia da Dronetech, de 2016 (cortesia do Serviço de Comunicação Social, Museu Paraense Emílio Goeldi)

Para melhor explorar o assunto, que ainda não foi desenvolvido pela historiografia, embora seja fundamental para a história de um bem tombado pelo Instituto do Patrimônio Histórico e Artístico Nacional, dividimos este artigo em três tópicos. No primeiro, analisamos as razões que justificaram a aquisição de um imóvel relativamente distante do Centro da cidade de Belém para ali ser instalada a nova sede do Museu Paraense. No segundo tópico, foi analisada a primeira fase da expansão do museu, entre 1895 e 1908. No terceiro, a segunda fase, entre 1933 e 1943, já na Era Vargas (1930-1945). Nas considerações finais é apresentada uma tabela com a relação dos moradores expropriados e a localização de seus lotes, e também alguns dos possíveis desdobramentos deste estudo.

As fontes utilizadas na pesquisa consistem em documentos administrativos preservados no Arquivo Guilherme de La Penha, do Museu Paraense Emílio Goeldi, relatórios institucionais publicados por essa instituição e plantas baixas produzidas por funcionários do museu e do governo do estado, conservadas no Arquivo Público do Estado do Pará. Foram também de particular importância os relatórios, as mensagens governamentais e os diários oficiais do Pará, por nos ajudar a entender trâmites administrativos e jurídicos, como eram negociados os recursos junto ao Congresso Estadual para a desapropriação de terrenos e edificações, e para a construção de viveiros, recintos para animais, monumentos e lagos no parque zoobotânico.

Por fim, convém ressaltar a importância dos inventários *post-mortem*, preservados no Centro de Memória da Amazônia, da Universidade Federal do Pará, que ajudaram a identificar o perfil socioeconômico de alguns moradores cujos imóveis foram desapropriados. Essa documentação forneceu detalhes sobre os bens, o prestígio e as alianças familiares nas quais estavam envolvidos os inventariados, uma gama de informações que nos possibilitou reconstituir, embora parcialmente, a forma pela qual tais sujeitos negociaram e foram agentes no processo de desapropriação. Além disso, foram consultados jornais, leis e decretos, imagens impressas e fotografias preservados nas instituições mencionadas.

### O início da construção do parque zoobotânico

Em 1891, no contexto das primeiras reformas republicanas na instrução pública, o Museu Paraense foi instalado na rua São João, no Centro de Belém, ao lado do antigo Liceu Paraense (Sanjad, 2010). Sob a direção de Ernesto Acton, especialista em taxidermia, o museu recebia muitas doações de animais vivos, mantidos em condições precárias. Em 1893, Acton sugeriu ao governador Lauro Sodré a criação de um jardim botânico e de um zoológico. Esses espaços foram concretizados na gestão de Emílio Goeldi, iniciada em março de 1894. Em 2 de junho do mesmo ano, o novo regulamento do museu, decretado por Sodré, definiu a instalação do jardim zoológico e do horto botânico como “anexos” do museu (Regulamento..., 1894).

Para que esse projeto fosse realizável, Goeldi considerou fundamental encontrar um “edifício mais apropriado e com capacidade bastante para permitir o desenvolvimento e aumento das coleções”, uma vez que as dimensões e a precariedade do antigo prédio mostravam-se em desacordo com o que o governador pretendia para o “novo museu” (Goeldi, 1895, p.218). O diretor e seus encarregados, após terem examinado com atenção diversos imóveis da cidade, manifestaram grande interesse pela propriedade de Bento José da Silva Santos, dono da rocinha n.22, localizada na estrada da Independência (atual avenida Governador Magalhães Barata).

Tal como as chácaras, as rocinhas localizavam-se nos subúrbios, em terrenos amplos e ajardinados, onde também se cultivavam hortas e pomares e se criavam animais para consumo familiar desde finais do século XVIII. No auge da economia da borracha surgiram requintadas moradias, que passaram a aliar o conforto da vida urbana com a paisagem rural (Soares, 1996; Soares, 2008). Como havia interesse de Sodré e de Goeldi em adquirir a rocinha de Silva Santos, uma das maiores da cidade, o proprietário decidiu negociá-la por Rs.120:000$000 (contos de réis ouro), exigindo que lhe fosse entregue como adiantamento Rs.20:000$000 no ato da compra. O valor foi pago, ao passo que o montante da dívida foi negociado pelo erário estadual. Goeldi (1895, p.218) fez a seguinte observação sobre o imóvel:

Disposição arquitetônica interior, sólida estrutura, dimensões, conservação esmerada, aspecto ameno, foram outros tantos fatores de recomendação, visto que a rocinha acha-se dentro de terrenos não pequenos que permitem a realização d’aqueles anexos do Museu, que o governo tem em mente, a saber um modesto Jardim Zoológico e um Horto Botânico, ficou-se convencido que entre as propriedades públicas e privadas atualmente disponíveis na cidade nenhuma apresentava igual soma de vantagens e qualidades recomendáveis.

Convém lembrar que, a despeito das qualidades arquitetônicas do edifício, o local – “dentro de terrenos não pequenos” – rememorava uma antiga área de lazer de Belém, pois ali mesmo funcionara, na década de 1870, um jardim público. Esse jardim estendia-se da travessa Nove de Janeiro até a atual travessa Quatorze de Março, nos fundos da basílica de Nossa Senhora de Nazaré, tendo uma frente de mais de quinhentos metros. Tratava-se de um sítio privado, reservado para o divertimento do público visitante, do qual era cobrado o ingresso. As fontes consultadas sugerem que o espaço foi inaugurado, inicialmente, como Circo Olympico, em finais de 1870, sendo depois rebatizado, em 4 de junho de 1871, como Jardim Mitológico (O Liberal..., 20 jul. 1871; Diário de Belém, 15 out. 1882; Diário de Notícias, 4 dez. 1894, 7 dez. 1894). O lugar, contudo, funcionou por pouco tempo. Já no final da década de 1870, o imenso terreno foi desmembrado e vendido aos poucos pelos seus proprietários. Bento José da Silva Santos adquiriu seis lotes, construindo, em 1879, no maior deles, com frente para a estrada da Independência, a sua rocinha. Os demais lotes do antigo Jardim Mitológico foram adquiridos de formas variadas por outras pessoas.

A desapropriação da rocinha de Silva Santos e a construção dos “anexos” do Museu Paraense a partir de 1895 não tiveram relação direta com o Jardim Mitológico, e sim com a disponibilidade de terrenos oriundos do espólio do antigo proprietário desse jardim. Somente Silva Santos detinha, na década de 1890, 1/4 do quarteirão onde hoje se situa o parque zoobotânico Museu Goeldi. Portanto, a nova sede do museu não tentava rememorar a experiência do antigo jardim, mas se alinhava à política urbanística de Belém implementada a partir de 1890, principalmente o esforço para criar áreas verdes e espaços de lazer.

A Intendência de Belém participou diretamente do planejamento da nova sede do museu por meio da Sociedade Zeladora do Museu Paraense, criada em 1896 e presidida pelo intendente José Coelho da Gama e Abreu, o barão de Marajó. Essa sociedade tinha como objetivos proteger, conservar e promover o museu, tornando-o um centro de pesquisa e de instrução pública. Goeldi buscou o apoio da elite política local para concretizar seus planos, fazendo da Sociedade Zeladora um intermediário entre o museu, a Intendência, o governo e o Congresso Estadual (Goeldi, 1897a; Marajó, 1897).

A partir de 1895, a rocinha e os terrenos de Silva Santos passaram por uma radical transformação arquitetônica e paisagística, tendo em vista adaptar as instalações para que pudessem abrigar o acervo do museu e seu mobiliário (Suescun Florez, Sanjad, Okada, 2018). A área somava 14.962m^2^ e se estendia transversalmente, dividindo o quarteirão em duas partes, da estrada da Independência à estrada da Constituição (atual avenida Gentil Bittencourt), com a diferença de que a parte frontal era mais larga em relação aos fundos. No governo de Paes de Carvalho, os lotes de Silva Santos e todos os outros quatro lotes que existiam em direção à travessa Nove de Janeiro, todos edificados, foram declarados de utilidade pública pelo estado do Pará com base na lei 499, de 15 de maio de 1897. Isso equivalia à metade do quarteirão, como pode ser observado na planta baixa publicada por Goeldi em 1897 (Figura 2).

**Figura 2:** : Planta baixa do Museu Paraense de História Natural e Etnografia em 1896, onde é possível observar o imóvel desapropriado para a instalação do museu (em rosa) e as intervenções feitas e planejadas para esse espaço e também para os terrenos vizinhos em direção à travessa Nove de Janeiro (Goeldi, 1897b)

Os recursos aprovados a partir de 1896 pelo Congresso Estadual permitiram a realização de intervenções em todo o espaço, de maneira a organizar as exposições internas, os dois “anexos”, os laboratórios, as oficinas e as residências dos funcionários. Tanto o horto quanto o zoológico foram concebidos para fins de instrução pública, uma vez que se buscava criar uma “atraente escola de intuição das obras da natureza amazônica”, mas de proporções modestas, sem a intenção de “querer imitar os grandes jardins e hortos de além-mar” (Goeldi, 1895, p.220).

A transformação realizada nesse espaço, como a construção de jaulas, viveiros, aleias, canteiros, plantações, lagos, edificações e monumentos, já foi analisada pela historiografia, incluindo no que diz respeito ao plantel inicial e à primeira coleção vegetal ali reunida. Também já foi analisada a reação e a recepção positiva do público em relação à nova sede do Museu Paraense (Sanjad, 2006, 2008, 2009, 2010; Machado, 2010; Sanjad et al., 2012; Suescun Florez, 2015; Suescun Florez, Sanjad, Okada, 2018). O que importa ressaltar, no momento, é que as atribuições de Goeldi não se resumiam à concepção e à coordenação das obras executadas no parque. Coube a ele também negociar acordos com os moradores vizinhos à instituição, no sentido de alugar suas residências e lotes e de aceitar o montante que o governo oferecia a título de desapropriação. Segundo Goeldi (1897b, p.258-259), os contratos firmados com os moradores tinham o objetivo de impedir “oscilações arbitrárias”, ou seja, possíveis exigências ou pedidos de reajuste, os quais poderiam interferir no projeto de instalação do horto e do zoológico. Nesse sentido, os contratos pareciam atender aos interesses do governo e do museu, algumas vezes em oposição ao desejo dos moradores, como veremos adiante.

### A primeira fase de expansão do parque zoobotânico

Após a promulgação da lei 499, em 1897, a qual deu início ao processo de desapropriação dos lotes existentes em direção à travessa Nove de Janeiro (ver Figura 2), o governo estadual organizou uma comissão avaliadora composta pelos engenheiros Henrique Santa Rosa (diretor de Obras Públicas) e Victor Maria da Silva (inspetor das Águas de Belém), com a dupla função de examinar a conservação dos imóveis e determinar seu valor. Goeldi considerou deveras importante que essa comissão iniciasse suas inspeções pelas propriedades de Manoel Alves da Cruz (parcela I da planta baixa que aparece na Figura 2) e de João Ribeiro de Miranda (parcela III da mesma planta), haja vista que ambas ocupavam os espaços onde foi projetado o horto botânico. Havia, portanto, necessidade imediata da retirada desses moradores (Goeldi, 1897b, p.3).

Goeldi utilizou argumentos autoritários para justificar a desapropriação do imóvel de Manoel Alves da Cruz, alegando que ele se eximia de pactos e compromissos, fazia “exigências” e “cobranças de aluguéis exorbitantes” por uma rocinha em péssimo estado de conservação, com paredes “pensas e rachadas” e “assoalhos podres”, além de ser insalubre e anti-higiênica para quem não tivesse “constituição de batráquio” (Goeldi, 1897b, p.259; Goeldi, 1900, p.3-4). Na concepção do diretor do museu, o que, de fato, tinha valor era “o terreno e não a moradia”, uma vez que seria mais adequado demoli-la do que reformá-la (Goeldi, 1900, p.4).

Cruz não aceitou a proposta feita pelos engenheiros do governo para a desapropriação de sua rocinha, considerada por ele abaixo do valor de mercado. Entretanto, o prédio e seu terreno foram alugados ao Museu Paraense – e isso também foi motivo para litígios com o governo. Em agosto de 1898, o *Diário Oficial do Estado* publicou um ofício de Cruz pedindo que o procurador fiscal do Tesouro autorizasse o aumento do aluguel de sua propriedade, de Rs.200$000 para Rs.300$000 mensais, de maneira a equipará-lo com o valor pago pelo Museu Paraense por outros imóveis vizinhos (Diário Oficial…, 14 ago. 1898, p.326). Passados alguns dias, Cruz recebeu como resposta que nem o diretor do museu nem o governador do estado haviam autorizado o reajuste, pois a ação para a desapropriação do imóvel já estava “em juízo”, não convindo “aos interesses do Estado” a elevação do valor pago pelo imóvel (Diário Oficial…, 27 set. 1898, p.605).

Ao recusar a proposta feita pelos engenheiros do governo, Cruz acabou tendo que suportar a intolerância do poder público. Em março de 1900, ele recorreu ao secretário de estado, dessa vez pedindo que interferisse no conflito que travava com Goeldi. O contrato de aluguel havia finalizado e não fora renovado por Cruz, que solicitara a Goeldi a desocupação do imóvel e a devolução das chaves. Goeldi se recusou a entregar-lhe as chaves e Cruz enviou novo ofício, dessa vez ao governador Paes de Carvalho, pedindo-lhe que fosse devolvida sua casa para que ali fosse morar com sua família (Secretaria do Estado do Pará, 27 jul. 1900, p.1).

A estratégia de Goeldi era ganhar tempo até que a desapropriação fosse resolvida no âmbito judicial em favor do governo. Isso ocorreu somente em 1º de fevereiro de 1901, já na gestão de Augusto Montenegro, quando foi declarada a posse definitiva e imediata, pelo Museu Paraense, da rocinha de Manuel Alves da Cruz e do rancho de João Miranda de Ribeiro (Montenegro, 1901). Contudo, a posse do imóvel de Cruz foi efetivada somente em 28 de agosto de 1901, após Virgílio Cardoso de Oliveira, secretário de Estado da Justiça, Interior e Instrução Pública, ter notificado o diretor do Museu Goeldi de que a casa já poderia ser utilizada (Secretaria de Estado da Justiça…, 28 ago. 1901).

Não se sabe, precisamente, o porquê do atraso, mas é possível deduzir que os recursos judiciais acionados por Cruz tenham prolongado um pouco mais o conflito, além dos protestos públicos que ele fazia na imprensa local contra a ação do governo. Cruz não recebeu sua indenização em dinheiro, mas sim em uma nota promissória do estado do Pará, no valor de Rs.28:500$000. Além de o valor ter sido contestado pelo proprietário, ele ainda sofreu recorrentes atrasos no pagamento dos aluguéis entre julho de 1898 e agosto de 1901. Cruz tornou pública sua insatisfação com a irregularidade nesses pagamentos, mas foi ignorado pelas autoridades do estado e pelo diretor do museu (Diário Oficial…, 8 jul. 1898, p.73, 25 ago. 1898, p.391, 10 jan. 1900, p.50, 16 mar. 1900, p.521, 6 abr. 1900, p.40, 9 maio 1900, p.229, 31 ago. 1901, p.299).

Por sua vez, Goeldi (1904, p.4) se disse “aliviado” com essa desapropriação, visto que a demora em finalizar o processo constituiu uma “fonte de contrariedades e aborrecimentos”. Embora o dito prédio não tivesse serventia para os planos de Goeldi, o terreno onde ele se encontrava era de suma importância para a instalação do horto botânico. Uma vez obtido o lote, o prédio foi demolido, e, no local, foi construída uma caixa d’água cenográfica, conhecida como Castelinho, e instalados diversos viveiros de plantas e gaiolas para aves. Na horta que ficava nos fundos, foi feita uma plantação de árvores produtoras de látex, isto é, o espaço foi reaproveitado pela equipe da Seção de Botânica para a musealização de espécimes vivos (Huber, 1901, p.104).

Quanto ao rancho de João Ribeiro de Miranda, localizado na travessa Nove de Janeiro, n.34, o proprietário aceitou a avaliação dos peritos, restando apenas o trâmite processual para que o acordo firmado por ambas as partes pudesse ser reconhecido juridicamente. Porém, Goeldi (1901, p.106-107) recusou-se a aguardar os trâmites formais, pois receava que as coisas “viessem a complicar-se”, recomendando ao governo que a desapropriação daquele imóvel fosse realizada com a “possível brevidade”. Goeldi tinha um interesse particular em adquirir aquele reduzido terreno de 560m^2^, ocupado por uma singela taberna, onde, possivelmente, eram vendidas bebidas alcoólicas baratas. Para Goeldi (1904, p.4), a taberna tinha um aspecto desagradável – “repugnante esteticamente” – e constituía-se em “foco de desmoralização”, prejudicando a disciplina interna que ele impôs aos funcionários do museu, principalmente aos jardineiros e tratadores de animais. A casa foi descrita como estando “em ruínas” e avaliada em Rs.8:000$000 pelos peritos do governo.

O rancho propriamente dito era, segundo Goeldi (1906, p.469), “uma vacaria sita no antigo capinzal”, o que nos permite entrever o uso dado àquele terreno. Possivelmente, tratava-se de uma antiga propriedade para a criação de vacas-leiteiras, uma atividade produtiva bastante comum na periferia de Belém naquela época. Ribeiro de Miranda, falecido logo depois da desapropriação, deixou como herança à viúva Antônia Maria de Jesus a indenização a que tinha direito (Inventário de João..., 27 set. 1898). Sua taberna foi logo eliminada, dando lugar à residência do inspetor do horto botânico, Andreas Goeldi, primo de Emílio, construída no estilo de um chalé suíço; e ainda um depósito de vidros e materiais utilizados na conservação de coleções e também uma moradia para os serventes do museu (Goeldi, 1906, p. 469). Essa mudança traria enormes benefícios para a instituição, pois a casa do inspetor assumiria uma posição estratégica nesse espaço, satisfazendo a necessidade de fiscalização do lado oriental do horto e funcionando como portaria para o controle do acesso de serviço (Goeldi, 1904, p.9).

Para efeito de comparação, convém analisar o processo de desapropriação dos lotes de Bento José da Silva Santos. As fontes elucidam que ele foi um importante empresário da segunda metade do século XIX, cujos negócios combinavam atividades agrícolas, extrativistas, comerciais e industriais em Chaves, na ilha de Marajó, em municípios da costa paraense, na ilha de Arapiranga (defronte a Belém, mas pertencente ao município de Barcarena) e em Belém. De acordo com o inventário *post-mortem* de Silva Santos, a empresa da família, Silva Santos & Filhos, era composta pelos seguintes ativos: 12 terrenos e imóveis em Belém, fazendas, benfeitorias, criação de gado, trapiches, barcos e uma lancha a vapor, maquinários e utensílios, além de investimentos no plantio de seringueiras. Falecido em 23 de maio de 1908, Silva Santos legou um patrimônio imobiliário contabilizado em 539:000$000 (contos de réis), sem contabilizar os bens da empresa (Inventário de Bento..., 23 maio 1908).

Silva Santos, assim como muitos outros seringalistas, pecuaristas e industriais, ocupou diversos cargos públicos, condição necessária para a reprodução de sua fortuna e também para fazer alianças familiares com outros proprietários. Fazia-se necessário aliar a riqueza a outros fatores, como nome e sobrenome, sobretudo por meio das alianças conjugais (Cunha, 2008). Por exemplo, Silva Santos casou sua filha Maria da Silva Santos com o deputado Pedro Leite Chermont, pertencente à família que dominou a política local no século XIX e também na Primeira República. Cancela (2011, p.353) esclarece que alianças desse tipo muitas vezes “funcionavam como estratégia de conservação do patrimônio, assim como de restrição à entrada de indivíduos sem linhagem na rede de parentesco”. Tais estratégias, restritas ao universo da elite paraense, pretendiam também perpetuar o sobrenome das famílias imbricadas.

Na época da desapropriação dos seus terrenos localizados na estrada da Independência e na travessa Nove de Janeiro, Silva Santos fazia parte do Conselho Municipal, onde permaneceu por muito tempo. O ano de 1895 marca a cessão de sua rocinha para a instalação do Museu Paraense de História Natural e Etnografia e, coincidentemente, o início da construção de um palacete e das casas de seus filhos casados em quatro lotes que ele possuía na estrada de Nazaré, nas proximidades da praça da República. O palacete Silva Santos, depois renomeado palacete Faciola, hoje sede do Museu da Imagem e do Som, é um dos edifícios icônicos construídos pela elite que controlava os meios de produção e os serviços públicos em Belém no final do século XIX (Derenji, 2023; Marques, Baleixe, 2023).

Diferentemente de Manoel Alves da Cruz e de João Ribeiro de Miranda, pode-se inferir que Silva Santos tinha uma posição privilegiada para definir, de acordo com suas conveniências, o preço do aluguel de seus imóveis e o valor atribuído a eles. Isso pode ser comprovado por meio de um telegrama expedido pelo vogal à Secretaria do Estado, em novembro de 1898, declarando que desejava elevar o valor do aluguel de um de seus terrenos cedidos ao Museu Paraense de Rs.300$000 para Rs.400$000 mensais (Diário Oficial…, 4 nov. 1898, p.197). Em 19 de julho de 1899, Augusto Olímpio de Souza, secretário do estado, informou a Goeldi que, mediante uma “comunicação” feita por Silva Santos, resolvera reajustar o aluguel de suas propriedades para Rs.400$000 mensais, retroativos a 1º de janeiro daquele ano, “até que seja realizada a desapropriação já autorizada” (Secretaria do Estado do Pará, 19 jul. 1899).

O interessante é que o mesmo pedido havia sido feito – e negado pelo governo – por Manoel Alves da Cruz, três meses antes de Silva Santos. O argumento para o indeferimento do pedido de Cruz foi exatamente o mesmo utilizado para o deferimento do pedido de Silva Santos. Enquanto, para o primeiro, o reajuste não seria concedido porque o imóvel estava em processo de desapropriação, para o segundo, o reajuste era justificável porque o imóvel seria desapropriado. Como se observa, o tratamento dado a Silva Santos foi diferente, mesmo estando o seu imóvel em situação similar ao de Cruz, ou seja, em processo de desapropriação.

O contraste entre ambos – Cruz e Silva Santos – não findou aí. Enquanto Cruz recebeu uma nota promissória pela indenização de seu imóvel, Silva Santos, em 30 de agosto de 1899, mediante autorização do Tesouro Público, recebeu a quantia de Rs.26:000$000, seis a mais do que o estipulado no acordo inicial (Diário Oficial…, 8 jan. 1899, p.45). Em seguida, Silva Santos colocou à venda, ao governo do estado, seus outros lotes vizinhos à rocinha de número 22, por Rs.32:000$000. A proposta foi aceita de imediato pelo governo, sem negociação. No dia 22 de setembro de 1899, menos de um mês depois do primeiro pagamento que lhe fora concedido, o Museu Paraense entrava com a posse definitiva de todos os lotes que haviam pertencido a Silva Santos (Goeldi, 1901, p.106-107).

Depois de os lotes da travessa Nove de Janeiro terem sido integrados ao Museu Paraense, foram dados os passos iniciais para que pudessem ser incorporados os imóveis da estrada da Constituição. Destarte, pelo ofício 483, de 27 de novembro de 1899, foi declarado ao Tesouro Público o aluguel da casa n.123, de propriedade de Diogo Pinto da Silva, pelo preço mensal de Rs.250$000 (lote VI da Figura 2, que, em 1897, tinha como proprietário Domingos da Motta Nogueira) (Secretaria do Estado do Pará, 27 nov. 1899). Contudo, decorridas algumas semanas, o governo sancionou o decreto 794, de 5 de janeiro de 1900, autorizando o Tesouro a abrir um crédito extra de Rs.8:880$208 para que fosse realizada a desapropriação dessa mesma casa (Diário Oficial…, 5 jan. 1900, p.30). O morador foi comunicado pessoalmente por Goeldi, em 10 de janeiro de 1900, e consentiu em desocupar o imóvel para que nele fossem instalados alguns pesquisadores do museu.

Oito anos depois, mais uma casa na estrada da Constituição foi adquirida pelo governador Montenegro, pelo valor de Rs.20:000$000. Era pertencente à viúva e aos órfãos Maia (lote V da Figura 2). No entanto, pelo que nos foi dado saber, esse mesmo lote, anos antes, fora oferecido ao governo pela quantia de Rs.70:000$000, sendo a proposta recusada, uma vez que o valor foi considerado exorbitante. O processo de negociação prolongou-se durante muitos anos e foi finalizado somente em 1908, com um valor bem mais baixo do que o pretendido pela proprietária. Esse foi o último lote a ser incorporado ao museu no perímetro entre a rocinha de Silva Santos e a travessa Nove de Janeiro (Montenegro, 1908, p.175).

Mesmo tendo sido realizadas importantes obras no espaço museal desde 1897, Goeldi queixava-se recorrentemente da falta de espaço. Segundo ele, o Museu Paraense já necessitava, em 1899, em caráter de urgência, de um “segundo edifício igual ou maior do que o existente”, visto que os salões e corredores do prédio central já estavam abarrotados com as coleções. A construção de mais um edifício seria a chave para resolver parte dos problemas que atormentavam o diretor. Com essa justificativa, foi solicitada ao Congresso Estadual a aquisição do restante do quarteirão situado em direção à travessa Vinte e Dois de Junho (atual avenida Alcindo Cacela), especialmente de uma casa pertencente ao médico Miguel José de Almeida Pernambuco, visto que ali Goeldi pretendia abrigar as seções de Botânica e Geologia (Goeldi, 1901, p.107-108).

O processo de aquisição da residência de Miguel Pernambuco deu início à expansão do museu em direção à travessa Vinte e Dois de Junho. Apesar de o pedido inicial de Goeldi ter sido feito no início de 1900 por meio de um ofício ao inspetor do Tesouro Estadual (Diário Oficial..., 14 jan. 1900, p.80), somente a partir de 1903 o governador Montenegro (1902, p.40) conseguiu os recursos necessários para iniciar essa nova fase.

Em 1903, a rocinha de Hermínio Cardozo da Cunha Coimbra, localizada na estrada da Independência, n.42, ao lado da rocinha de Silva Santos, também foi negociada pelo museu. Essa rocinha foi deixada a Hermínio como herança de sua mãe, Libéria Maria Joaquina Corrêa Coimbra, falecida em 1900 (Inventário de Libéria..., 17 out. 1900). Libéria havia sido casada com o comerciante português Antônio Cardoso da Cunha Coimbra e com ele teve três filhos: José, Hermínio e Joaquim Cardoso da Cunha Coimbra. José, o primogênito, foi nomeado representante legal da família para fazer a partilha dos bens deixados em testamento, sob o consentimento de seu pai, que ainda estava vivo. Hermínio, que morava em Taubaté-SP, negociou a parte que lhe coube com o museu quando estava de passagem por Belém em 1903, recebendo o valor de Rs.12:600$000 somente em 1905. Os outros dois filhos, José e Joaquim, também herdaram terrenos naquele mesmo quarteirão, mas que só seriam desapropriados décadas mais tarde, como veremos no próximo tópico.

Em uma planta de 1907, elaborada por Jacques Huber, sucessor de Goeldi na direção do museu, e conservada no Arquivo Público do Estado do Pará, vê-se o projeto paisagístico detalhado do jardim que deveria ser construído na área da antiga rocinha de Hermínio, incluindo canteiros, aleias e monumentos (Figura 3). A planta também mostra as espécies vegetais a ser introduzidas e como o novo espaço foi integrado à área existente do parque. Esse novo jardim foi inaugurado em 1908, juntamente com o busto do fundador do museu, o naturalista mineiro Domingos Soares Ferreira Penna (1818-1888), esculpido por Rodolpho Bernardelli (Sanjad, 2010).

**Figura 3:** : Projeto paisagístico do jardim construído sobre a rocinha de Hermínio Cardozo da Cunha Coimbra (em rosa), de autoria de Jacques Huber, 1907 (Arquivo Público do Estado do Pará, Belém)

Essa contínua expansão do parque demonstra o apoio que a instituição recebeu do governo estadual ao longo de três gestões ou 15 anos, que liberava anualmente um crédito suplementar de Rs.30:000$000 para a sua ampliação e melhorias, elevado a Rs.50:000$000 em 1901, equivalente à metade do orçamento anual da instituição (Goeldi, 1904). Nesse período, o Museu Paraense foi a instituição pública que mais recebeu investimentos do governo estadual (Sanjad, 2010).

### A segunda fase de expansão do parque zoobotânico

Em 1901, Aristides Pereira Leão, agrimensor da Secretaria de Obras Públicas do governo estadual, fez um levantamento fundiário e topográfico do quarteirão onde o Museu Paraense estava sendo instalado, a pedido de Goeldi, em escala 1:400 (Figura 4). Essa planta está preservada no Arquivo Público do Estado do Pará e, assim como a da Figura 3, permaneceu desconhecida até a realização desta pesquisa. Ela documenta as intervenções na quadra desde a inauguração do parque em 1895 e a delimitação dos lotes e residências em direção à travessa Vinte e Dois de Junho, ausente na planta publicada por Goeldi em 1897 (ver Figura 2). Devido ao péssimo estado de conservação da planta de 1901, o documento foi recriado digitalmente para que pudesse ser analisado.

**Figura 4:** : Planta vetorizada do parque zoobotânico do Museu Paraense de História Natural e Etnografia (PA) em 1901, elaborada a partir do original desenhado pelo agrimensor Aristides Pereira Leão, preservado no Arquivo Público do Estado do Pará, Belém. Essa planta permite visualizar a segunda etapa do processo de expansão do parque (elaborada por Fernando Carlos, em 2020, utilizando o *software* ArcGis 10.7)

Os lotes já incorporados ao patrimônio do museu em 1901 aparecem em cinza-claro na Figura 4. No canto superior direito, o imóvel da viúva Maia, desapropriado em 1897, só foi adquirido em 1908. Os lotes na direção da travessa Vinte e Dois de Junho, em branco, ainda eram particulares, incluindo a rocinha e os terrenos da família Cunha Coimbra. Em rosa, aparece a localização da rocinha de Hermínio Cardozo da Cunha Coimbra, negociada em 1903 e adquirida em 1905. Convém observar que o lote onde ficava essa rocinha era apenas uma pequena fração do patrimônio fundiário da família naquele quarteirão.

A aquisição dos lotes localizados em direção à travessa Vinte e Dois de Junho só se efetivou na gestão de Carlos Estevão de Oliveira (1930-1945), durante a ditadura de Getúlio Vargas. Embora Oliveira tenha levado adiante um plano traçado por Goeldi no final do século XIX, o contexto político era diferente. As primeiras desapropriações para a instalação do parque zoobotânico foram todas custeadas pelo governo estadual, que também financiou a transformação do espaço museal por meio da contrução de canteiros, recintos para animais, monumentos, lagos e edificações. Conforme Sanjad (2010), esse processo integrou o conjunto de reformas educacionais e urbanísticas implementado pelos primeiros governos republicanos do Pará. O Museu Paraense foi considerado, simultaneamente, um recurso pedagógico destinado a formar cidadãos, uma evidência do estado de civilização da elite local e um instrumento para a propaganda de Belém, cidade em grande projeção internacional devido ao comércio da borracha.

Na Era Vargas, o Museu Paraense foi ressignificado conforme o discurso modernista e nacionalista que caracterizou o período. Figueiredo (2001) analisou o assunto chamando a atenção para a expansão das políticas públicas do governo federal e para as alianças de Vargas com os grupos políticos locais, processo que novamente inseriu o Museu Paraense no centro das atenções políticas. O projeto institucional de Goeldi, jogado no limbo da administração pública paraense nos anos 1920, quando a crise da economia da borracha foi mais intensa, foi recuperado e redimensionado para as prioridades estabelecidas por Vargas e seus acólitos locais. Neste artigo, não cabe analisar toda a complexidade do assunto, que pode ser mais bem compreendido nos trabalhos de Leal (2023, 2024), sobre a articulação feita por Oliveira para a inserção do Museu Paraense em políticas públicas nacionais, e Andrade (2024), sobre os discursos formulados por Vargas para a Amazônia e as intervenções estatais no território regional, sobretudo nos campos da saúde e da nutrição.

O que importa ressaltar é que o momento mostrou-se propício para a consecução dos antigos planos de Goeldi: expandir o parque zoobotânico até a travessa Vinte e Dois de Junho e garantir apoio para a ampliação da infraestrutura institucional, não mais às custas do falido governo estadual, mas do federal, que se mostrou receptivo às demandas regionais e disposto a investimentos em ciência e tecnologia. Em 1930, ainda restavam oito lotes a ser incorporados ao patrimônio do museu: quatro pertencentes aos irmãos José e Joaquim da Cunha Coimbra, dois pertencentes a Suzana Evangelina Autran, um de Felippe Lima e um de Agostinho de Menezes Monteiro (ver Figura 4).

Carlos Estevão de Oliveira retomou o processo de desapropriação dos lotes vizinhos ao museu em 1933, depois que o governo federal fez um convênio com o estado do Pará visando ao repasse de recursos financeiros, destinados à expansão do horto botânico, à instalação de um orquidário e de um bosque de palmeiras (Palmarum) e à construção de um laboratório de ictiologia e de tanques para a criação de peixes. Esses dois últimos itens eram demandas do próprio governo federal, que pretendia inserir o Museu Paraense no Programa Nacional de Incentivo à Piscicultura, na época coordenado pela Comissão Técnica de Piscicultura do Nordeste. Esse programa priorizou o investimento em pesquisas sobre a reprodução de peixes amazônicos para o povoamento dos açudes nordestinos, aliando políticas de ciência e alimentação com obras contra as secas (Oliveira, 2022; Leal, 2024).

Em razão desse projeto estratégico, o governo estadual desapropriou as duas casas pertencentes à viúva Suzana Autran. No início do século, havia um único imóvel, mas ela o dividiu em dois após a morte do marido, Agostinho Autran, passando a residir em uma casa e alugando a outra (Inventário de Agostinho..., 27 jan. 1933). Suzana, no mesmo ano em que recebeu a herança, foi pressionada a negociá-la com o governo do Pará, pois a área foi considerada de utilidade pública para anexação ao Museu Paraense. O valor pago à viúva foi de Rs.16:000$000 (Inventário de Agostinho..., 27 jan. 1933). No local das residências foram construídos o Palmarum e o orquidário, inaugurados em 1934 (Dyer, 1945, p.3).

Nesse mesmo ano, outros lotes conjugados foram adquiridos com verbas arrecadadas pelo Museu Paraense com o comércio de peixes ornamentais amazônicos (Leal, 2024). Os lotes eram de propriedade da família Cunha Coimbra e de Felippe Lima, localizados na esquina da travessa Vinte e Dois de Junho com a avenida Gentil Bittencourt (antiga estrada da Constituição) (Suescun Florez, 2015). Nesses terrenos teve início a construção do campo de piscicultura do museu, projeto que tinha como objetivo ampliar a “capacidade da seção zoológica” (Barata, 1944, p.172).

Essas aquisições deram ao museu maior controle sobre o quarteirão, embora ainda restassem por desapropriar o grande terreno da família Cunha Coimbra e o sobrado do médico Agostinho de Menezes Monteiro, com frente para a avenida da Independência (antiga estrada da Independência), marcado com um asterisco na planta de 1901 (Figura 4) porque ainda não havia sido construído naquela época. Os recursos do governo federal permitiram a aquisição destes últimos lotes. Entre 1935 e 1941, o governo federal concedeu cinco subvenções ao museu, totalizando Cr$510.000,00 (quinhentos e dez mil cruzeiros), dos quais Cr$320.000,00 (trezentos e vinte mil cruzeiros) foram investidos apenas em desapropriações, conforme os valores e a moeda adotada em 1942 (Museu Paraense..., 11 nov. 1942, 14 nov. 1942).

Foi Agostinho Monteiro quem protagonizou o último (e tenso) processo de desapropriação. Conforme Castro (13 jun. 2013), ele foi médico, professor e político. Administrou o Hospital da Ordem Terceira de São Francisco e a Faculdade de Medicina e Cirurgia do Pará. Lecionou na Escola Prática do Comércio e na Escola de Agronomia e Veterinária do Pará. Foi deputado estadual de 1926 a 1930 e deputado federal em 1934 pela Frente Única Paraense, que fazia oposição ao grupo político do interventor Magalhães Barata. No início da década de 1940, Monteiro adquiriu o grande terreno pertencente a José e Joaquim da Cunha Coimbra. O plano de Monteiro era construir, nessa extensa área, um prédio horizontal com dois pavimentos e 48 apartamentos. Esse conjunto residencial, pensado e elaborado pelo arquiteto J.E. Levi, teria um jardim com aleias e palmeiras nas áreas centrais e laterais. O financiamento da obra foi negociado com a Caixa Econômica Federal, com o Instituto de Aposentadoria e o Lar Brasileiro. No início de 1941, restava apenas a assinatura do prefeito de Belém, Abelardo Conduru, para que fosse expedido o despacho que autorizaria o início das obras. Se construído, o Edifício Marajó tomaria quase 1/3 do quarteirão onde hoje se encontra o parque zoobotânico (Folha do Norte, 16 abr. 1941, p.2, 26 abr. 1941, p.2).

Carlos Estêvão conseguiu impedir a construção do prédio, anunciada por Monteiro nos jornais de Belém, ao enviar um parecer técnico a José Malcher, interventor do estado. Em 31 de março de 1941, a interventoria embargou a obra planejada por Monteiro sob o argumento de que o prédio impediria a expansão do museu. Um decreto determinou que os imóveis na avenida da Independência e na travessa Vinte e Dois de Junho, que ainda não pertenciam ao Museu Paraense, deveriam ser negociados com o estado mediante indenização, cujo valor seria determinado após avaliação técnica. O prefeito, acatando a decisão do interventor, informou Monteiro que não poderia autorizar o projeto, pois o governo do estado havia decretado a expropriação do imóvel para a ampliação do museu (Folha do Norte, 16 abr. 1941, p.2).

Em 12 de maio de 1941, Malcher autorizou a desapropriação do sobrado de Agostinho Monteiro e do grande terreno com frente para a travessa Vinte e Dois de Junho. O planejado condomínio permaneceu apenas no papel, levando Monteiro a negociar o terreno com o governo estadual, mas adquirido com verbas federais. Ali foram instalados laboratórios, lagos e tanques para a criação intensiva de várias espécies de quelônios e peixes amazônicos, inaugurados em 1943 e integrados às ações da Comissão Técnica de Piscicultura do Nordeste, além de abrigos temporários para peixes ornamentais, na época comercializados pelo museu (Leal, 2024). Quanto ao sobrado com frente para a avenida da Independência, foi preservado pela administração do museu, assim como a residência vizinha do médico Miguel de Almeida Pernambuco. Esses dois imóveis, juntamente com a rocinha de Silva Santos e uma outra casa localizada na travessa Nove de Janeiro, n.40 (lote IVd na planta publicada em 1897, ver Figura 2), utilizada como residência dos diretores do museu até a década de 1950, são as únicas edificações remanescentes dos antigos moradores daquele quarteirão.

## Considerações finais

Durante as gestões de Emílio Goeldi (1894-1907), Jacques Huber (1907-1914) e Carlos Estêvão de Oliveira (1930-1945) foram desapropriados vinte lotes, pertencentes a 11 pessoas ou famílias, para a construção do parque zoobotânico. Esse processo começou em 1895 e terminou em 1941, quando todos os lotes foram incorporados ao patrimônio do museu. Na gestão de Goeldi, ocorreram 11 desapropriações e aquisições entre 1895 e 1905; na de Huber, houve apenas uma aquisição em 1908, de um imóvel desapropriado em 1897; na de Carlos Estêvão, houve oito desapropriações e aquisições entre 1933 e 1941. Bento José da Silva Santos e Agostinho Monteiro foram os maiores proprietários desapropriados, com seis e três lotes, respectivamente.

No Quadro 1 é possível visualizar a relação de proprietários, a localização dos terrenos desapropriados e a data da aquisição, que não deve ser confundida com a data da desapropriação, pois a essa se seguia um período de negociação quanto ao valor a ser pago pelo governo estadual, período que poderia se estender por muitos anos. A elaboração dessa tabela exigiu o acompanhamento de cada etapa de todos os processos de desapropriação, investigação executada em diversos documentos, como os boletins do Museu Paraense, o *Diário Oficial do Estado*, inventários *post-mortem*, jornais e plantas baixas.

**Quadro 1:** : Relação de proprietários e de imóveis ou terrenos desapropriados para a construção do parque zoobotânico do Museu Paraense Emílio Goeldi entre 1895 e 1941

Proprietários	Localização	N. de lotes	Data da aquisição
Bento José da Silva Santos	Estrada da Independência, n.22 Estrada da Independência, s.n. Travessa Nove de Janeiro, s.n. Travessa Nove de Janeiro, s.n. Travessa Nove de Janeiro, s.n. Travessa Nove de Janeiro, n.40	6	1895-1899
Diogo Pinto da Silva (identificado como sendo de Domingos da Mota Nogueira em 1897)	Estrada da Constituição, n.123	1	1899
Manoel Alves da Cruz	Estrada da Independência, n.24	1	1901
João Ribeiro de Miranda	Travessa Nove de Janeiro, n.34	1	1901
Miguel José de Almeida Pernambuco	Estrada da Independência, ?	1	1903-1905
Herminio Cardozo da Cunha Coimbra	Estrada da Independência, n.42	1	1905
Viúva e órfãos Maia	Estrada da Constituição, n.125	1	1908
Suzana Evangelina Autran	Travessa Vinte e Dois de Junho, n.439 Travessa Vinte e Dois de Junho, n.445	2	1933
José e Joaquim Cardozo da Cunha Coimbra	Travessa Vinte e Dois de Junho, ?	2	1934
Felippe J. Lima	Estrada da Constituição, ?	1	c. 1941
Agostinho Menezes Monteiro, Julia Menezes Monteiro e herdeiros de Maria Dias Brito	Estrada da Independência, n.172 Travessa Vinte e Dois de Junho, n.182-184 Travessa Vinte e Dois de Junho, n.186	3	1941

Fonte: elaborado pelos autores.

Na primeira fase de expansão do museu, moradores que resistiram ou recusaram as ofertas por seus imóveis foram estigmatizados publicamente por Goeldi, que os considerava “obstáculos ao progresso” e usava termos depreciativos para descrever suas residências. Goeldi tinha uma visão preconceituosa sobre os imóveis mais modestos e o modo de vida local. Não é possível afirmar se Huber compartilhava dessas ideias, pois ele não expressou opiniões similares em seus relatórios. Embora Huber também defendesse mais desapropriações para ampliar o horto botânico, ele foi menos incisivo com os vizinhos do museu. A suspensão das desapropriações durante sua gestão deve-se aos baixos investimentos no museu após 1910, devido às oscilações negativas no mercado da borracha, o que resultou em problemas administrativos, atraso de salários, redução do orçamento e do número de funcionários, além da diminuição no número de expedições, de publicações e na aquisição de coleções científicas (Sanjad, 2019).

A paralisação do programa de desapropriações também pode ser explicada pela valorização dos terrenos vizinhos ao museu, consequência direta das melhorias na infraestrutura urbana demandadas pelo próprio museu, como o calçamento e a pavimentação das ruas do entorno, o acesso à água encanada, a instalação da rede elétrica e a vigilância noturna, todos aspectos que mereceram comentários de Goeldi em seus relatórios anuais. De maneira geral, os imóveis do bairro encareceram, tornando o programa de desapropriações muito oneroso para o orçamento do estado. As últimas desapropriações só foram realizadas graças aos recursos federais transferidos no governo de Getúlio Vargas e intermediados por Carlos Estevão de Oliveira.

Goeldi, Huber e Oliveira justificaram a expansão do Museu Paraense em razão do espaço necessário ao acondicionamento de coleções e da ampliação da infraestrutura de pesquisa, como escritórios e laboratórios. Eles também desqualificaram os moradores vizinhos e defenderam a intervenção estatal como necessária para criar um espaço de instrução e sociabilidade em Belém. O alto número de visitantes no parque, assunto abordado por Sanjad (2006, 2010), foi central na retórica dos governadores do estado e diretores do museu, sendo usado para legitimar as intervenções no quarteirão, obter recursos do Congresso Estadual e angariar apoio político durante as negociações com os moradores, que muitas vezes se tornaram públicas. Por outro lado, o apoio político e financeiro que o museu recebeu no primeiro governo de Getúlio Vargas foi o elemento catalisador para que as desapropriações fossem retomadas, após um intervalo de 25 anos sem investimentos do governo estadual. O envolvimento do museu (e de Carlos Estêvão) em projetos e ações governamentais de âmbito federal na região amazônica, como os levados pela Comissão Técnica de Piscicultura do Nordeste, pelo Serviço do Patrimônio Histórico e Artístico Nacional e pelo Conselho de Fiscalização das Expedições Artísticas e Científicas no Brasil, garantiu à instituição uma relativa estabilidade na burocracia do governo estadual, se compararmos os anos 1930-1940 com a década de 1920 (Leal, 2023, 2024).

O Museu Paraense, evidentemente, não foi a única instituição brasileira transformada ou criada com o advento da República. A historiografia vem analisando esse processo em diversas instituições científicas longevas, como a Escola de Minas de Ouro Preto (Carvalho, 1978), o Museu Nacional (Lopes, 1997), o Museu Paulista (Alves, 2001), o Jardim Botânico do Rio de Janeiro (Bediaga et al., 2008; Heizer, 2012), o Instituto Oswaldo Cruz (Benchimol, 1990), além de sociedades médico-científicas e faculdades de medicina. Heizer e Videira (2010) organizaram um volume para discutir trajetórias profissionais e as transformações provocadas nas instituições de ensino e pesquisa pelo regime republicano. Os estudos, contudo, raramente se dedicam à análise da inserção dessas instituições na cidade. Poderíamos perguntar, por exemplo, qual o impacto provocado no bairro de São Cristóvão com a transferência do Museu Nacional para o Palácio da Boa Vista em 1892; ou como a instalação do Museu Paulista no Monumento do Ipiranga, a partir de 1893, afetou a vida da população no entorno. Se quisermos extrapolar essas perguntas para períodos posteriores, seria lícito questionar sobre as consequências das intervenções urbanas que deram origem ao Museu Histórico Nacional, em 1922, e da criação do Museu Imperial em Petrópolis, em 1940.

Os estudos no campo da saúde e medicina parecem dedicar mais atenção à relação entre o processo de institucionalização da ciência e seus impactos na vida urbana, como comprovam as pesquisas de Benchimol (1999), Almeida (2003) e Mota (2005). Foi essa a abordagem priorizada nesta pesquisa: compreender, em uma microescala, como as transformações nos campos político e científico afetaram a vida das pessoas que residiam no entorno da sede do Museu Paraense a partir de 1895. Essas pessoas foram diretamente impactadas pelas decisões de Lauro Sodré e Emílio Goeldi. Ambos se empenharam em criar uma instituição modelar, com forte carga simbólica para as elites políticas locais e, de modo simultâneo, fortemente conectada às redes científicas internacionais, como já demonstrou a historiografia (Lopes, 1997; Sanjad, 2010). Este artigo evidencia que, além de uma abordagem mais institucional, focalizando o organograma, o orçamento, a produção científica, os funcionários, os intercâmbios nacionais e internacionais, as relações com o governo etc., outro olhar é possível, preocupado com o coletivo social fisicamente conectado com a instituição e por ela permanentemente afetado.

## Data Availability

Não estão em repositório.
